# NK Cytotoxicity Mediated by NK-92 Cell Lines Expressing Combinations of Two Allelic Variants for *FCGR3*

**DOI:** 10.3390/antib13030055

**Published:** 2024-07-12

**Authors:** Marta Freitas Monteiro, Maria Papaserafeim, Matteo Andreani, Aline Réal, Athanasios Kouklas, Daniela Reis Galvão, Jörg D. Seebach, Gisella L. Puga Yung

**Affiliations:** Laboratory of Translational Immunology, Department of Medicine, Division of Immunology and Allergology, University Hospitals Geneva, Medical Faculty, CH-1211 Geneva, Switzerland

**Keywords:** NK-92, *FCGR3A*, CD16, ADCC, L66H/R, V176F, L48H/R, V158F

## Abstract

Natural killer (NK) cells play an important role in the surveillance of viral infections and cancer. NK cell antibody-dependent cellular cytotoxicity (ADCC) and direct cytotoxicity are mediated by the recognition of antibody-coated target cells through the Fc gamma receptor IIIA (FcγRIIIa/CD16) and by ligands of activating/inhibitory NK receptors, respectively. Allelic variants of the *FCGR3A* gene include the high-affinity single-nucleotide polymorphism (SNP) rs396991 (V176F), which is associated with the efficacy of monoclonal antibody (mAb) therapies, and the SNP rs10127939 (L66H/R). The contribution of *FCGR3A* SNPs to NK cell effector functions remains controversial; therefore, we generated a panel of eight NK-92 cell lines expressing specific combinations of these SNPs and tested their cytotoxicities. NK-92 cells were stably transfected with plasmids containing different combinations of *FCGR3A* SNPs. Messenger RNA and FcγRIIIa/CD16 cell surface expressions were detected using new generation sequencing (NGS) and flow cytometry, respectively. All FcγRIIIa/CD16-transfected NK-92 cell lines exhibited robust ADCC against three different target cell lines with minor differences. In addition, enhanced direct NK cytotoxicity against K562 target cells was observed, suggesting a mechanistic role of FcγRIIIa/CD16 in direct NK cytotoxicity. In conclusion, we generated eight FcγRIIIa/CD16-transfected NK-92 cell lines carrying different combinations of two of the most studied *FCGR3A* SNPs, representing the major genotypes described in the European population. The functional characterization of these cell lines revealed differences in ADCC and direct NK cytotoxicity that may have implications for the design of adoptive cancer immunotherapies using NK cells and tumor antigen-directed mAbs.

## 1. Introduction

Natural killer (NK) cells are a subpopulation of lymphocytes that play an important role in the immune response to viral infections and in cancer immunosurveillance [1,2,3,4]. The cardinal effector functions of NK cells are (i) the lysis of transformed (stressed) target cells triggered by the predominant expression of ligands for activating NK receptors, leading to “natural” or “direct” NK cytotoxicity; (ii) the induction of apoptosis through death receptors; and (iii) the recognition of antibody-coated target cells through their Fc gamma receptor IIIA (FcγRIIIa/CD16), leading to antibody-dependent cellular cytotoxicity (ADCC) [4]. In addition to the level of CD56 expression, the presence of FcγRIIIa/CD16 on the cell surface allows for the discrimination between NK subpopulations that are highly cytotoxic (CD56^dim^CD16^pos^) or predominantly cytokine-producing NK cells (CD56^bright^CD16^dim/neg^) [1,5].

The FcγRIIIa/CD16 receptor consists of two extracellular Ig-like domains, D1 and D2; a stalk region; a transmembrane domain; and a short cytoplasmic tail and has a low affinity for immunoglobulin G (IgG) subclasses compared to other Fc-gamma receptors [6]. The Fc fragments of IgGs bind to the FcγRIIIa/CD16 at the beginning of D2 near the hinge region between D1 and D2 [7,8,9]. In addition to NK cells, subsets of monocytes and macrophages and small subpopulations of dendritic cells and T cells express FcγRIIIa/CD16 on their surface [10,11,12]. FcγRIIIa/CD16 has different affinities for the different IgG subclasses [9] and, upon activation, is either internalized for recycling or cleaved by ADAM17 (“a disintegrin and metalloprotease 17”), which cleaves the stalk region and thereby reduces NK cytotoxicity [13,14].

Several single nucleotide polymorphisms (SNPs) have been described for *FCGR3A*. The most studied SNP is the rs396991 (V176F) located in D2, which affects the binding of IgG to the receptor [15,16]. Furthermore, the tri-allelic SNP rs10127939 (L66H/R) in D1 is relevant for NK cytotoxicity; it influences the binding of IgG subclasses to the receptor, with H having a higher binding to human IgG1, IgG3, and IgG4 than L [17]. However, the consequences of combinations of these two SNP in the context of human disease and intravenous immunoglobulin G and mAb therapies remain to be investigated [18,19,20,21,22,23,24]. 

Therapeutic mAbs are engineered to target specific molecules. EGFR is a transmembrane protein, a member of a large family of receptors involved in developmental biology and cancer. EGFR overexpression, mutation, or both contribute to the malignant phenotype of many cancer cells. Thus, the design of therapeutic mAbs, such as cetuximab, that target EGFR has led to improvements in the outcome of cancer patients [25]. Rituximab and Obinutuzumab are IgG1 subclass mAbs that bind to CD20, a transmembrane protein expressed on B cells. Although the exact biological function and physiological ligand remain elusive, CD20 plays a major role in regulating B-cell activation and proliferation by acting as a Ca^2+^ channel. There are two types of anti-CD20 mAbs based on their ability or inability to translocate CD20 to lipid rafts after binding, type-I and -II, respectively [26]. Clinically, anti-CD20 mAb therapies are largely used to treat B-cell-derived hematologic malignancies, autoimmune diseases, and humoral transplant rejection. Since the approval of rituximab [27] and several other therapeutic antibodies, it remains controversial whether or not the clinical efficacy of mAb therapy is associated with the *FCGR3A* rs396991 (V176F) SNP [28,29,30,31,32,33,34,35,36,37,38,39]. The clinical consequences of rs10127939 and rs396991 combinations in the context of human disease and intravenous immunoglobulin G and mAb therapies remain to be investigated [18,21,22,23,24,25,26]. Furthermore, *FCGR3A* SNPs are clinically relevant in infections such as malaria, severe COVID-19, bacteremia in transplant patients, periodontitis, as well as in Kaposi’s sarcoma in HIV-infected patients and autoimmune diseases, including lupus and rheumatoid arthritis [21,40,41,42].

The CD3^neg^CD56^pos^ human NK-92 cell line, characterized by a large granular lymphocytic phenotype, was originally obtained from a patient with non-Hodgkin’s lymphoma [43,44]. Cell culture of NK-92 cells is dependent on high doses of interleukin-2 (IL2), and the function and phenotype resemble activated NK cells with granzyme- and perforin-containing granules and the surface expression of CD2, CD7, CD25, CD69, NKp30, NKp46, and CD94 [43,44,45]. In contrast, NK-92 cells lack FcγRIIIa/CD16 expression and, consequently, do not mediate ADCC [46]. To evaluate ADCC in vitro, NK-92 cells have been transfected with murine CD16 and high-affinity CD16 CAR in the past [47,48] and transduced with low-affinity CD16 to test therapeutic mAbs [49]. 

The aim of the present study was to generate and compare transfectants derived from the NK-92 cell line carrying different combinations of the rs10127939 (L66H/R) and rs396991 (V176F) *FCGR3A* polymorphisms. In particular, the effect of specific SNP combinations on NK cytotoxicity, both ADCC and direct killing, was investigated.

## 2. Materials and Methods

### 2.1. Reagents

*Biologicals*: The human type-II anti-CD20 antibody (Obinutuzumab, non-glycoengineered) used in this study was kindly provided by Christian Klein from Roche Innovation Center, Zurich. Human anti-EGFR (anti-EGFR-IgG1) antibody was purchased from Invivogen (San Diego, CA, USA). Human recombinant IL2 was obtained from Novartis Pharma (Proleukin, Basel, Switzerland).

*Antibodies*: Three different antibody clones against FcγRIIIa/CD16 (B73.1, 3G8, and MEM154), anti-HLA-ABC, and anti-CD58 were used for flow cytometry. Mouse IgG1 and IgG2a isotype controls were also included. All antibodies were conjugated to either fluorescein isothiocyanate (FITC) or to R-phycoerythrin (PE). Additionally, F(ab’)_2_ fragment goat anti-human IgG, F(ab’)_2_ fragment specific (BCR blocking), goat anti-human κ F(ab’)_2_-PE, and goat polyclonal isotype control-PE were used (Table S1).

*Chemicals, culture media, and supplements*: Probenecid, bovine serum albumin (BSA), ethylenediaminetetraacetic acid (EDTA), and fetal bovine serum (FBS) were purchased from Sigma-Aldrich (St. Louis, MO, USA). Buffers Dulbecco’s Phosphate-Buffered Saline (PBS), Dulbecco’s Modified Eagle Medium (DMEM), Roswell Park Memorial Institute 1640 (RPMI), Minimum Essential Medium Amino Acids Solution (EAA), Non-Essential Amino Acids Solution (NEAA), 4-(2-hydroxyethyl)-1-piperazineethanesulfonic acid (HEPES), penicillin/streptomycin (Pen/Strep), sodium pyruvate, and geneticin were all from Gibco (Grand Island, NY, USA). The L-Alanyl-L-Glutamine (L-Glu/L-Ala) was from Bioswisstec AG, Schaffhausen, Switzerland. AB serum was obtained from the *“Centre de Transfusion de Genève”* of our institution, (Table S1).

*Cell lines, PBMC isolation, and culture conditions*: The cell lines A-431, Daudi, K562, Raji, and NK-92 were acquired from ATCC. (Manassas, VA, USA) NK-92 transduced with low-affinity CD16, with F in amino acid position 176, was a kind gift from Kerry Campbell and NantKwest (noGFP-CD16 176F NK-92.05, US patent No. 7,618,817; EU patent No. 1771471). Of note, positions 66 and 176 correspond to 48 and 158, respectively, depending on whether the leader peptide is considered (Table S1).

The A431 culture medium consisted of DMEM supplemented with 20 mM HEPES, 10% FCS, 1 mM sodium pyruvate, 2 mM L-Glu/L-Ala, 100 IU/mL of 1× NEAA and 1× EAA, and 100 µg/mL Pen/Strep. The Daudi, K562, and Raji culture media consisted of RPMI-1640 containing 25 mM HEPES, 10% FCS, 2 mM L-Glu/L-Ala, and 100 µg/mL Pen/Strep. NK-92 cells and the FcγRIIIa/CD16 stable transfectants were maintained in RPMI-1640 medium containing 25 mM HEPES, 15% FCS, 5% AB serum, 2 mM L-Glu/L-Ala, 1 mM sodium pyruvate, Pen/Strep (10,000 U/mL), and 100 IU/mL of IL2. In all cases, cells were maintained in 5% CO_2_ humidified incubator at 37 °C. In the case of ADCC assays, NK-92 cells were cultured without IL2 for 16 h to reduce direct cytotoxicity.

Human NK cells were isolated from the blood of healthy donors. Peripheral blood mononuclear cells (PBMCs) were separated using Ficoll-Paque PLUS density gradient centrifugation GE Healthcare (Uppsala, Sweden) centrifugation at 900× *g* for 20 min at room temperature without a brake. The cells were then washed twice in PBS and stored in cold FACS buffer (PBS with 0.1% BSA) before antibody staining for flow cytometry analysis.

### 2.2. Construction of Plasmids Carrying FCGR3A Allelic Variants and NK-92 Transfectant Cell Lines

The gene source for generating the different *FCGR3A* gene variants was the commercial vector GenScriptpUC19-Amp *FCGR3A* transcript variant 3 (NM_001127593.1) (GenScript, Piscataway, NJ, USA). *FCGR3A* was subcloned into the multiple cloning site 2 (mcs2) of the pVITRO-neo-mcs plasmid (InvivoGen, San Diego, CA, USA) through the ligation of PCR products obtained via amplification using the polymerase Q5 high-fidelity DNA polymerase (New England BioLabs, NEB, Ipswich, MA, USA). We attempted to use the mcs1 and mcs2 to generate “heterozygous” combinations of *FCGR3A*, but this proved impossible due to constant deletions, rearrangements, or aberrations after the transformation of the constructs into *E. coli*. Detailed information on the primers used can be found in Table S2. The PCR conditions consisted of 1 cycle of denaturation at 98 °C for 30 s, 30 cycles consisting of denaturation at 98 °C for 10 s, annealing at 71 °C for 30 s, and extension at 72 °C for 30 s, ending with a final extension at 72 °C for 2 min. After amplification, DNA was purified using a Monarch PCR & DNA Cleanup Kit (NEB). pVITRO-neo-mcs and PCR products were digested in a 25 μL reaction with high-fidelity AgeI and NheI (NEB) and ligated with Instant Sticky-end Ligase master mix (NEB) in a vector-to-insert ratio of 1:3. The resulting 5 μL reaction was transformed into 50 μL of chemically competent NEB 5-alpha competent *E. coli* (high efficiency, NEB by propagation; all products details in Table S1).

To generate the *FGCR3A* variants conferred by the rs10127939 (L66H/R) and rs396991 (V176F) SNPs, site-directed mutagenesis was performed using the Q5 Site-Directed Mutagenesis kit (Promega, Madison, WI, USA) in the subcloned pVITRO-neo-CD16A_VL_MCS2_ b vector with the primers described in Table S2 and according to the manufacturer’s specifications. All the PCR conditions for the site-directed mutagenesis were similar: 1 cycle at 98 °C for 30 s; 25 cycles of denaturation at 98 °C for 30 s, annealing at 66 °C for 30 s, and extension at 72 °C for 2 min; ending with 1 cycle at 72 °C for 3 min 36 s. The 5 μL of the resulting reaction kinase, ligase, and DpnI mix, which contained the vector, was transformed into 50 μL of chemically competent NEB 5-alpha Competent *E. coli* for propagation according to the manufacturer’s instructions and cultured for 16 h on Luria broth agar plates with 100 μg/mL ampicillin (Sigma-Aldrich). Large amounts of plasmids were recovered using the classic phenol/chloroform method [50]. Finally, Sanger sequencing (Microsynth AG, Balgach, SG, Switzerland) was performed at all steps to confirm that the constructs were as expected.

To generate the NK-92 transfectants, 2 μg of pVITRO-neo-mcs plasmids carrying the elongation factor 1 alpha (EF-1α) promoter, which allows for the stable and long-term expression of the genes in transfected cells, were used. Subsequently, 4 × 10^6^ NK-92 cells were transfected in the presence of 100 μL of Solution R from the Amaxa Cell Line Nucleofector Kit R via electroporation using the program A-24 of the Nucleofector I device according to the manufacturer’s instructions (Lonza AG, Stein, Switzerland). Since the pVITRO-neo-mcs plasmid contains a neomycin resistance gene, 600 μg/mL of geneticin was added 48 h after electroporation and maintained along with the cultures to maintain the selection pressure. The control conditions included cells electroporated with empty pVITRO-neo-mcs and pulsed only. All cells were maintained in a 5% CO_2_ humidified incubator at 37 °C. Single plasmid transfections were performed to obtain single *FCGR3A* variants (LL_VV, LL_FF, RR_VV, and HH_VV), while co-transfections were required to obtain allelic *FGCR3A* variants.

### 2.3. Determination of FCGR3A Variants in NK-92 Transfectants from Messenger RNA Followed by New-Generation Sequencing (NGS) Quantification

To detect the expression levels of the different *FCGR3A* variants in the NK-92 transfectants, total RNA was extracted using Trizol Reagent (Invitrogen, Carlsbad, CA, USA) according to the manufacturer’s instructions. The cDNA synthesis was conducted using a ProtoScript II First Strand cDNA Synthesis Kit according to the manufacturer’s instructions, starting with 0.5 μg of total RNA and 6 μM Random Primer Mix (both NEB) in a 20 μL final volume. The controls included tubes containing all RT reagents minus the RNA template or reverse transcriptase, designated “NAC-RT” and “no-RT” controls, respectively.

For cDNA synthesis, 1.25 μL of the cDNA reaction was used in a 25 μL reaction containing 0.5 U of Q5 High-Fidelity (NEB); 1.5 mM MgCl_2_ (Promega, Madison, WI, USA); 0.2 mM Deoxynucleotide (dNTP) Solution Mix (NEB); 0.2 μM of each primer (designated Seq 48_158 fw and Seq 48_158 rev, Microsynth AG, Balgach, Switzerland, Table S2); and in 1 × Q5 reaction buffer (NEB). The cDNA amplification PCR consisted of a denaturation step at 95 °C for 30 s; followed by 30 cycles of denaturation at 98 °C for 5 s, annealing at 65 °C for 20 s, and extension at 72 °C for 15 s; ending with a final extension at 72 °C for 2 min. Negative PCR controls consisted of the following: all PCR reagents plus (i) “NAC-RT control”, (ii) “no-RT control”, and (iii) minus the cDNA template, designated “NAC RT-PCR”, “no-RT/ PCR”, and “NAC-PCR” controls, respectively.

The PCR products were run on 1.2% agarose gels at a constant 75 volts for 1 h, and bands were visualized after 30 min of staining with a solution consisting of a 1:3000 dilution in water of GelRed Nucleic Acid Gel Stain (Biotium Inc., Fremont, CA, USA) and 0.1 M NaCl for 30 min according to the manufacturer’s instructions. The 100 bp ladder (ThermoScientific, Waltham, MA, USA) was used to determine DNA size.

PCR products were purified using Monarch PCR & DNA Cleanup Kit (NEB), resuspended in nuclease-free H_2_O, and quantified using the Qubit 4 fluorometer (ThermoFisher Scientific, Waltham, MA, USA) coupled to the Qubit 1× dsDNA High Sensitivity (HS) kit according to the manufacturer’s instructions. Next-generation sequencing (NGS) was performed at the iGE3 Genomics Platform (University of Geneva, Geneva, Switzerland). Briefly, libraries were generated using 100 ng of each PCR product and the TruSeq Nano DNA High Throughput Library Prep kit (Illumina, San Diego, CA, USA). Libraries were pooled at equimolar proportions (10 nM) before hybridization to a NovaSeq 6000 (Illumina) flow cell and paired-end 100 bp (PE100) sequencing methods. The quantification of *FCGR3A* variant expression was performed using the iGE3 Bioinformatics Platform (University of Geneva). Briefly, an average of approximately 10 million paired-end reads were obtained for each sample. Sequencing quality control (QC) was performed using the FastQC analysis tool [v0.12.1]. The reads were then aligned to the Ensembl GRCh38 genome reference using STAR v.2.7.4. The average alignment rate was 99.2%. The different genotypes at the SNP positions rs10127939 and rs396991 were then assessed using igvtools (IGV v.2.16.2). Additional alignment analysis was performed using the igvtools command “*samtools-flagstat*”, and no “*QC failed*” flagged reads were identified during the alignment analysis. For all sequencing samples in the dataset, 100% of the reads were correctly paired, and there were no singletons (i.e., reads with a sequence present exactly once, which is unique among the reads). This confirmed that all paired-end reads originated from the same DNA molecule and that, therefore, the identified SNPs were correctly matched.

### 2.4. Surface Marker Expression Using Flow Cytometry Analysis, Cell Sorting, and Cytotoxicity Assays with NK-92 Cell Lines

Direct labeling was used to assess the cell surface expression of FcγRIIIa/CD16 on NK-92 transfectants using three different mAb clones (3G8, B73.1, and MEM154), while HLA-ABC and CD58 expressions were assessed on targets cells. Cells were washed with FACS buffer and incubated for 30 min at 4 °C with saturating amounts of antibodies or isotype-matched controls. After the labeling, excess antibody was removed by washing with FACS buffer. To test for CD20 and EGFR antigen expressions on target cells, indirect labeling was required, starting with the B-cell receptor blockade using 0.4 mg/mL F(ab’)_2_ fragment goat anti-human IgG for 30 min at 4 °C. After incubation with anti-CD20 or anti-EGFR at concentrations ranging from 0.125 to 20 μg/mL, a secondary polyclonal antibody, goat anti-human κ F(ab’)_2_-PE, was used for final labeling. Data acquisition and analysis were performed using an Attune NxT Acoustic Focusing Cytometer (ThermoFisher Scientific) and FlowJo version 10.5.3 software (RRID:SCR_008520), respectively, using the gating strategy depicted in Figure S1. The surface expression levels were compared by calculating the geometric mean fluorescence intensity ratios (MFIRs) as previously described [51]. 

NK-92 transfectants were sorted for FcγRIIIa/CD16 expression using a MoFlo Astrios cell sorter (Beckman Coulter Life Sciences, Brea, CA, USA) at our institution’s flow cytometry core facility. Briefly, 20 × 10^6^ cells were stained with the mAb 3G8 and the appropriate isotype control antibody for 30 min at 4 °C, washed, and resuspended in 2 mL FACS buffer plus 2.5 mM EDTA. The 3G8-positive cell fraction was then collected, centrifuged, and seeded at 0.3 × 10^6^ cells/mL in the culture medium as previously described. Twenty-four hours after sorting, 600 µg/mL of geneticin was added to the cell cultures. The transfected NK-92 cells were cultured in the presence of 100 IU/mL of IL2.

### 2.5. Cytotoxicity Assays by NK-92 Cell Lines

Non-radioactive DELFIA EuTDA Cytotoxicity Reagents (PerkinElmer, Waltham, MA, USA) were used to determine the level of NK-92 cell-mediated cytotoxicity according to the manufacturer’s instructions and as previously described [52]. Briefly, the target cells (K562, Daudi, Raji, and A431) were labeled with BATDA for 20 min and washed with PBS containing 20 mM HEPES and 2.5 mM probenecid to inhibit dye leakage.

For the ADCC assays, NK-92 cell lines were deprived of IL2 overnight, washed, and maintained in a serum-free AIM-V medium. Fixed effector-to-target (E:T) ratios of 5:1 and 10:1 were used for the anti-CD20 and -EGFR dose-response assays, respectively. Direct cytotoxicity was assessed in all ADCC experiments by co-culturing the target cells and NK-92 effector cells in the absence of mAbs. The plates were incubated for 1 h when co-cultured with Daudi cells or 2 h for the other target cells at 5% CO_2_ and 37 °C.

For direct cytotoxicity assays against K562 targets, NK-92 cell lines were maintained in culture medium with 200 IU/mL IL2 and used at E:T ratios of 20:1; 10:1; 5:1; and 2.5:1. 

In both assays, the maximum release was obtained by the lysis of labeled target cells (Lysis Buffer, PerkinElmer). To assess cytotoxicity following co-incubation, the released TDA ligand in the supernatants was chelated with europium salt and measured in a time-resolved fluorometer (EnVision 2014 Multilabel reader, PerkinElmer). Cytotoxicity was expressed as a percentage (%) of specific lysis.

For the ADCC dose-response assays, the curves were fitted using the three-parameter dose-response curves for the agonist model. The agonist was the mAb, anti-CD20 or -EGFR, and the EC_50_ was estimated accordingly, where EC_50_ was the concentration at which the mAb produced a half-maximum response. In direct cytotoxicity assays, plots were fitted using the asymptote-modified exponential growth model of Pross et al. [53] to obtain the lytic units, defined as the number of effector cells required to lyse a given number of target cells. We defined the reference lytic level as 30% (LU_30_) for 10^7^ effector cells. The EC_50_ and LU_30_ were estimated, and all curves were plotted using Prism GraphPad (version 9.5.0, RRID:SCR_002798).

### 2.6. Statistical Analysis

Analysis was performed with GraphPad Version 10.2.0. Comparisons of paired determinations were performed using a ratio-paired *t*-test or mixed-effect analysis with Šídák’s multiple comparisons test. For unmatched determinations, the Brown–Forsythe and Welch ANOVA with Dunnett’s T3 multiple comparisons test correction or one-way ANOVA corrected by Tukey’s multiple comparisons test were used to compare among cell lines. Other statistic tests are indicated in the text. The correlation between the 3G8 MFIR and EC_50_ was analyzed by calculating Spearman’s Rank Correlation Coefficient. The statistical test used is indicated in each figure legend. The significance is indicated by the *p*-values in the graphs.

## 3. Results

### 3.1. Expression of mRNA of FCGR3A SNPs rs10127939 and rs396991 in Transfected NK-92 Cell Lines

The vector design for the *FCGR3A* SNP variants, rs10127939 (L66H/R) and rs396991 (V176F), started from the *FCGR3A* transcript variant 3 (NM_001127593.1). Sequence analysis confirmed that this plasmid contains the bases encoding the V and L for rs396991 and rs10127939, respectively (sequence provided in Figure S2). After initial subcloning into the multiple cloning site 2 of the pVITRO-neo-mcs, only the pVITROneoCD16A_VL_MCS2_ clone b was kept and used as the starting material for the site-directed mutagenesis. Thus, pVITROneoCD16A_VH_MCS2_ clones a/c, pVITROneoCD16A_VR_MCS2_ clones a/b/c, and pVITROneoCD16A_FL_MCS2_ clones 4/13/14/15 were generated and sequenced for quality control purposes. The constructs of all SNP variants for *FCGR3A* used in this study are shown in Table S3 and were used for the transfection of NK-92 cells.

Huang et al. previously reported that the NK-92 cell line carries the *FCGR3A* gene but fails to express it on the surface [46]. Moreover, the expression of transfected genes can be impaired either by de-novo methylation [54] or by their random integration into transcriptionally silent DNA regions [55]. Therefore, the expression of each *FCGR3A* transgene was assessed at the mRNA level using RT-PCR with primers designed to cover rs10127939 (L66H/R) and rs396991 (V176F), spanning exons 4 and 5 and excluding the <3.4 Kb intron 4. Deep sequencing of the RT-PCR amplicons allowed us to identify each variant and determine its frequency in NK-92 transfectants.

**Figure 1:**
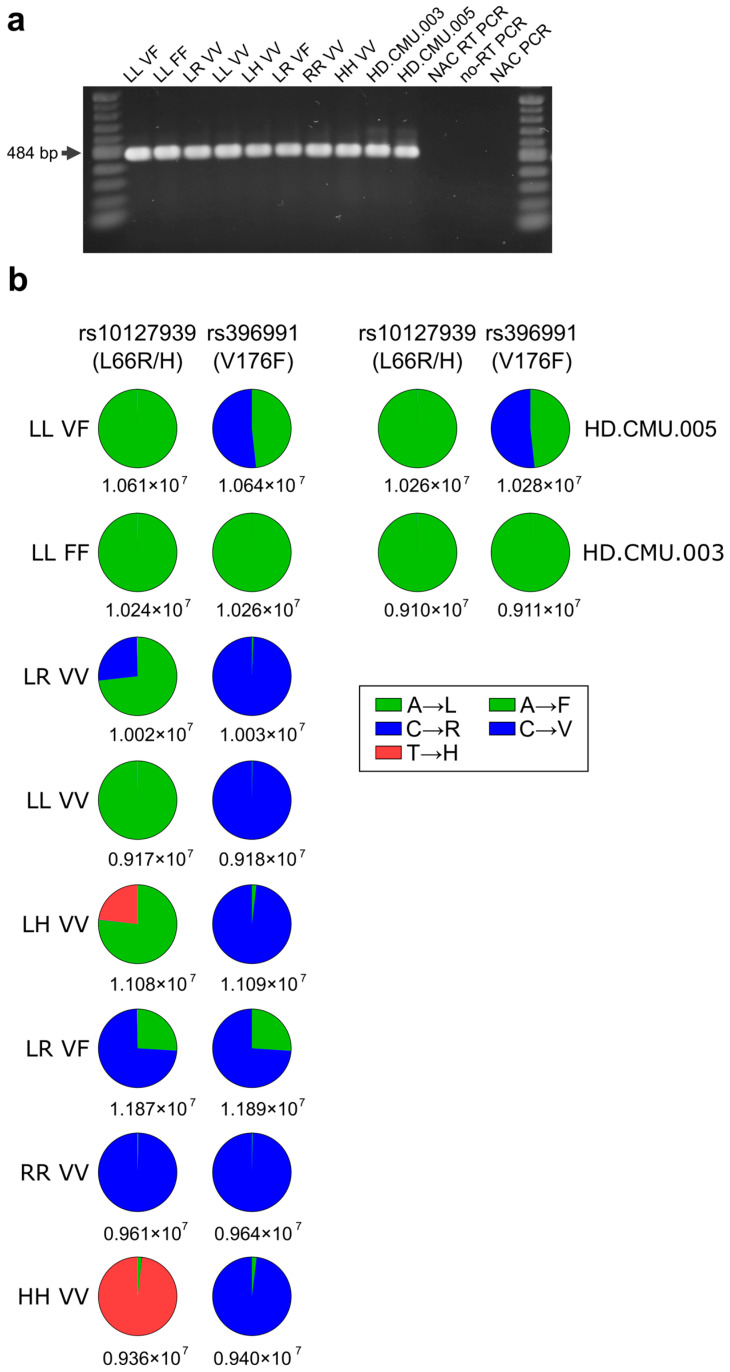
Messenger expression and NGS of NK-92 transfectants for the rs10127939 and rs396991 SNPs. (**a**) Total RNA was extracted from NK-92 transfectants and converted into cDNA. The cDNA was then amplified for a 484 bp region covering both SNPs, rs10127939 and rs396991. The resulting amplicons were run in a 1.2% agarose gel and stained with RedGel. These 484 bp fragments were used for the NGS analysis. The gel includes controls for cDNA synthesis and PCR, specifically the “No amplification controls” (NAC) for NAC RT-PCR and NAC PCR, as well as a control without reverse transcriptase (no-RT PCR). (**b**) The pie charts show the NGS reads corresponding to rs10127939 (L66H/R) and rs396991 (V176F) for each of the NK-92 transfectants. The total number of reads is shown below each pie chart, with bases represented by color codes. For each SNP, a specific base read corresponds to amino acids: leucine (L), arginine (R), histidine (H), phenylalanine (P), and valine (V).

The expression of a single *FCGR3A* variant was confirmed in the cell lines generated using single-plasmid transfection, namely NK-92 ^LL_FF^, NK-92 ^LL_VV^, NK-92 ^RR_VV^, and NK-92 ^HH_VV^. The expressions of two FCGR3A allelic variants were confirmed in NK-92 ^LL_VF^, NK-92 ^LR_VV^, NK-92 ^LH_VV^, and NK-92 ^LR_VF^. In the latter three cell lines, the ratio of the two transgene variants was approximately 1:3, possibly due to the intrinsic nature of random plasmid integration in the genome (Figure 1b). The results were confirmed via the sequencing of amplicons derived from the mRNA of two healthy human donors (HD.CMU.003, HD.CMU.005), whose genotypes have been previously confirmed using Sanger sequencing Figure S3.

In summary, the expression of either one or two sets of SNPs, rs10127939 (L66H/R) and rs396991 (V176F), in the eight different NK-92 cell lines generated in this study was confirmed through the NGS of amplicons derived from reverse-transcribed mRNA.

### 3.2. Stable FcγRIIIa/CD16 Cell Surface Expression of Different Combinations of the FCGR3A SNPs rs10127939 and rs396991

**Figure 2:**
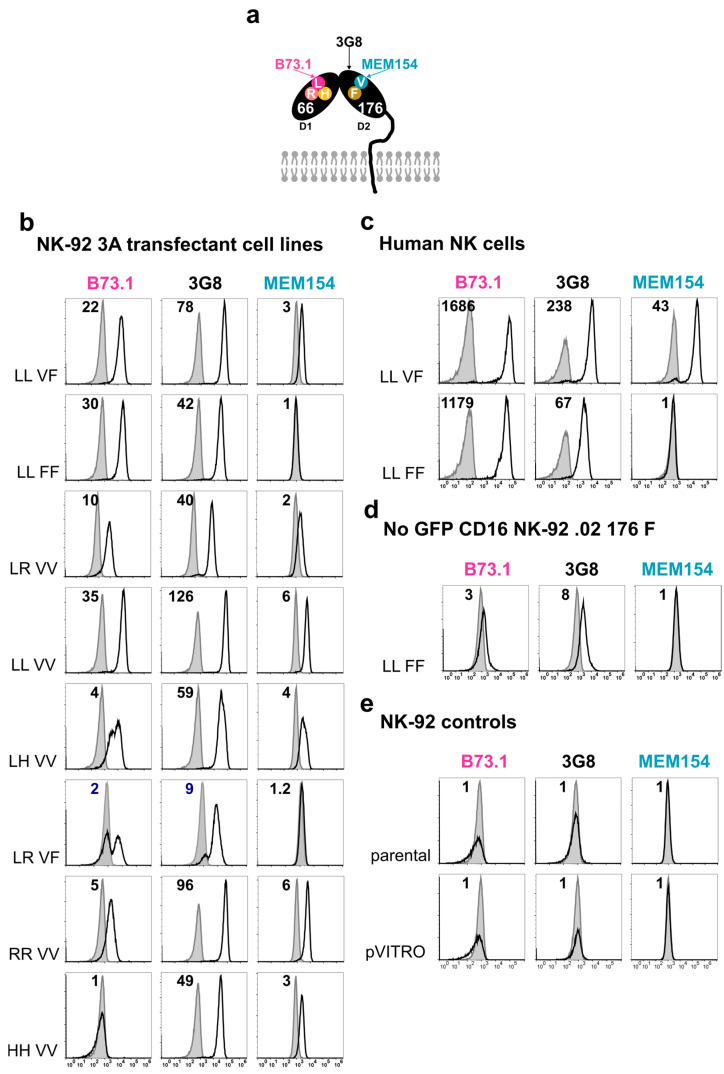

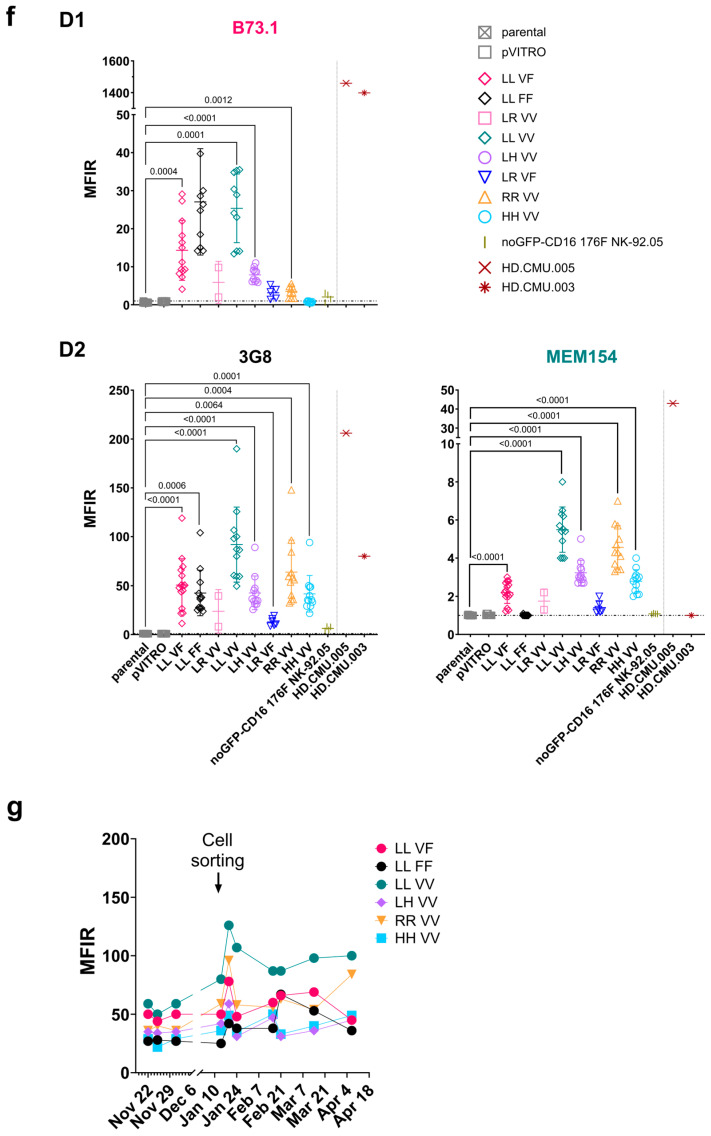
**Surface expression of FcγRIIIa/CD16 on NK-92 transfectants using different anti-CD16 monoclonal antibody clones.** Transfected NK-92 cell lines were tested for the surface expression of FcγRIIIa/CD16 using the mAb clones B73.1, 3G8, and MEM154 and matching isotype controls (grey), followed by flow cytometry analysis. (**a**) Schematic overview of the epitopes recognized by the anti-CD16 monoclonal antibody clones. These cover the different L66H/R and V176F variants of the FcγRIIIa/CD16 receptor located in domain 1 (D1) and domain 2 (D2), respectively. MEM154 only recognizes valine (V) at position 176 but not phenylalanine (F), whereas the clone B73.1 recognizes leucine (L) at position 66, binds poorly to arginine (R), but does not recognize histidine (H). In the case of clone 3G8, it recognizes a different epitope of D1, independent of the polymorphisms studied. (**b**–**e**) Representative histograms showing the recognition of FcγRIIIa/CD16 by the three clones. The L66H/R and V176F polymorphisms present in each cell line/donor are indicated next to the histograms, and the mean intensity ratio (MFIR) for each histogram is described in the upper-left corner. (**b**) The eight transfected cell lines (*n* = 10, except for NK-92 ^LL_VF^, NK-92 ^LR_VV^, and NK-92 ^LR_VF^, where n = 13, 2, and 5). (**c**) Single determination for the level of expression of FcγRIIIa/CD16 for two of the most common variants in fresh PBMCs gated on CD3^–^CD56^+^ NK cells are shown for donors HD.CMU.003 and HD.CMU.005. (**d**) Evaluation of FcγRIIIa/CD16 in the transduced cell line noGFP-CD16 176F NK-92.05 used as reference [49] (n = 3). (**e**) Transfection controls, including parental NK-92 and NK-92 ^pVITRO^ (n = 12 and 15, respectively). (**f**) Summary plots of FcγRIIIa/CD16 expression shown as MFIRs for the staining with anti-CD16 clones B731, 3G8, and MEM154 for all NK-92 cell lines (transfectants and transduced), including expression on fresh NK cells. One-way ANOVA (Brown–Forsythe and Welch) analysis for all cell lines except for human NK cells. (**g**) Time course of FcγRIIIa/CD16 expression as measured using 3G8 mAb FACS staining in six NK-92 transfectants over six months (80 passages), including a freeze/thaw cycle and cell sorting of the transfectants in January 2022 (arrow).

FcγRIIIa/CD16 cell surface expression of the eight NK-92 cell lines (Table S4), successfully transfected with different combinations of the SNP variants rs10127939 and rs396991 was determined using flow cytometry. Figure 2 shows the level of expression using three different mAb clones. Clone B73.1 binds to domain 1 (D1) of the FcγRIIIa/CD16 molecule, recognizing predominantly the L, weakly the R, but not the H variant; clone 3G8 binds to D2; and clone MEM154 has been reported to discriminate V from F by strong binding to V (Figure 2a) [15]. Figure 2b shows representative histograms for the relative expression of FcγRIIIa/CD16 compared to matched isotype controls when the transfectant cells were cultured in the presence of 100 IU/mL of IL2. Clone B73.1 showed high expression for transfectants with only L, i.e., NK-92 ^LL_VF^, NK-92 ^LL_FF^, and NK-92 ^LL_VV^ (MFIRs of 14.3 ± 7.9, 27.1 ± 14.0, and 25.4 ± 9.0, respectively), lower for the other combinations (MFIRs ranging from 3.2 ± 1.6 to 7.9 ± 1.9), and no staining for NK-92 ^HH_VV^ (MFIR of 0.8 ± 0.1; Table S5, left column). In our hands, MEM154 stained the NK-92 transfectants poorly (right column), with MFIRs ranging from 1.4 ± 0.3 to 5.5 ± 1.2 (Table S5, right column). In the case of clone 3G8, the MFIR varied between 13.0 ± 4.2 (NK-92 ^LR_VF^) and 91.9 ± 38.4 (NK-92 ^LL_VV^) (Table S5, middle column). Nonetheless, FcγRIIIa/CD16 expression was higher on freshly isolated NK cells compared to transfected NK-92 cells. Here, the clone MEM154 clearly discriminated between the donors with different V176F variants (Figure 2b,c). Our NK-92 ^LL_FF^ cell line showed 14- to 12-fold higher levels of FcγRIIIa/CD16 expression for B73 and 3G8, respectively, compared to the transduced noGFP-CD16 176F NK-92.05 cell line with the same combination of *FCGR3A* SNPs previously described by Binyamin et al. [49] (Figure 2b,c and Table S5). The parental NK-92 cell line, as well as NK-92 ^pVITRO^, had no detectable levels of FcγRIIIa/CD16 on its surface (Figure 2e,f). At last, FcγRIIIa/CD16 expression on NK-92 transfectants was stable after selection and cell sorting, as it did not change for more than five months or 80 cell culture passages (Figure 2g).

Thus, the eight transfected NK-92 cell lines not only express the different combinations of *FCGR3A* SNPs rs10127939 (L66H/R) and rs396991 (V176F) at the mRNA level, but also show stable surface expression.

**Figure 3:**
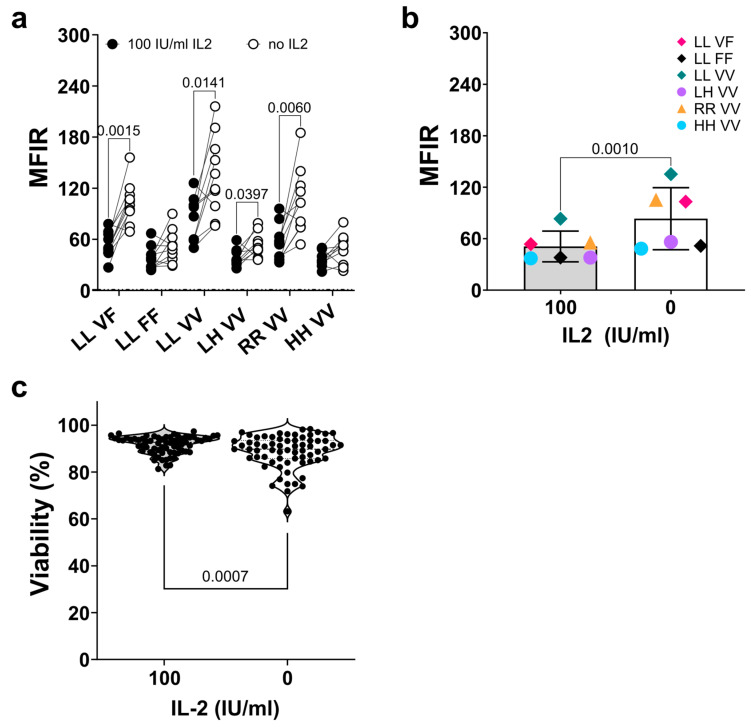
**Interleukin-2 deprivation increased FcγRIIIa/CD16 expression on NK-92 transfectants.** (**a**) Analysis of FcγRIIIa/CD16 expression using flow cytometry of the NK-92 cell lines stained with clone 3G8 after three days in culture with 100 IU/mL of IL2 (black circles) or IL2-deprived overnight (open circles) for six NK-92 transfectants. (**b**) Summary plot of the average of the mean fluorescence intensity ratio (MFIR) for each cell line analyzed under the two conditions previously described. In all cases, comparisons were made using a ratio-paired *t*-test, with n = 10. No values are shown if *p* ≥ 0.05. (**c**) Cell viability of NK-92 cell lines after short-term IL-2 deprivation. Different NK-92 cell lines, including both transfected and non-transfected cells, were cultured in the presence of IL-2 (100 IU/mL) or in the absence of IL-2 (0) for 16 h. Cell viability was analyzed using flow cytometry using the dead cell exclusion dye 7-AAD, and comparisons were made using a paired *t*-test, with n = 67.

The culture, proliferation, and cytotoxicity of the NK-92 cell line are IL2 dependent [43,44]. Nevertheless, to better distinguish between direct cytotoxicity and ADCC, we deprived NK-92 cells of IL2 prior to the assays [56,57]. It is also known that the activation of human NK cells leads to the membrane shedding of FcγRIIIa/CD16 by ADAM-17 (13, 14). Thus, it was of interest to determine whether overnight IL2 withdrawal from the cultures affected FcγRIIIa/CD16 surface expression in six of our NK-92 transfectants. Flow cytometric analysis using the 3G8 clone demonstrated that four out of the six transfectants tested (NK-92 ^LL_VF^, NK-92 ^LL_VV^, NK-92 ^LH_VV^, and NK-92 ^RR_VV^) exhibited increased FcγRIIIa/CD16 expressions when deprived of IL2 overnight, with adjusted *p*-values of 0.0015, 0.0141, 0.0397, and 0.0060, respectively (Figure 3a). In contrast, the two other cell lines, NK-92 ^LL_FF^ and NK-92 ^HH_VV^, did not significantly change their FcγRIIIa/CD16 expressions. NK-92 ^LL_VV^ showed the highest expression (3G8 MFIR values of 83 ± 24.0 and 135.2 ± 46.7, with and without IL2, respectively) and NK-92 ^HH_VV^ the lowest expression (37.1 ± 9.68 and 48.4 ± 17.6, with and without IL2, respectively). The pooled mean FcγRIIIa/CD16 expression of the six NK-92 transfectants increased upon IL2 deprivation (*p* = 0.0010), with variations from 1.3- to 2-fold higher MFIRs in the case of NK-92 ^HH_VV^, and NK-92 ^RR_VV^, or NK-92 ^LL_VF^ (Figure 3b). The same analysis, using clone MEM154, also revealed a significant increase in FcγRIIIa/CD16 expression upon IL2 deprivation for most of the cell lines, with the exception of NK-92 ^LL_FF^ and NK-92 ^HH_VV^. For the clone B73.1, only NK-92 ^LL_VF^ showed an increase in FcγRIIIa/CD16 (*p* = 0.0213) after IL2 deprivation (Figure S4). The overnight removal of IL2 resulted in a slight reduction in the viability of NK-92 cells, from 91.6 ± 4.4% to 88.7 ± 2.4%, which was nevertheless statistically significant (*p* = 0.0007). In all but one instance, the viability of the cells remained above 70% (Figure 3c).

In conclusion, overnight deprivation significantly increased the surface expression of FcγRIIIa/CD16 in the NK-92 transfectants.

### 3.3. Characterization of Cell Surface Antigen Expression on Target Cells

**Figure 4:**
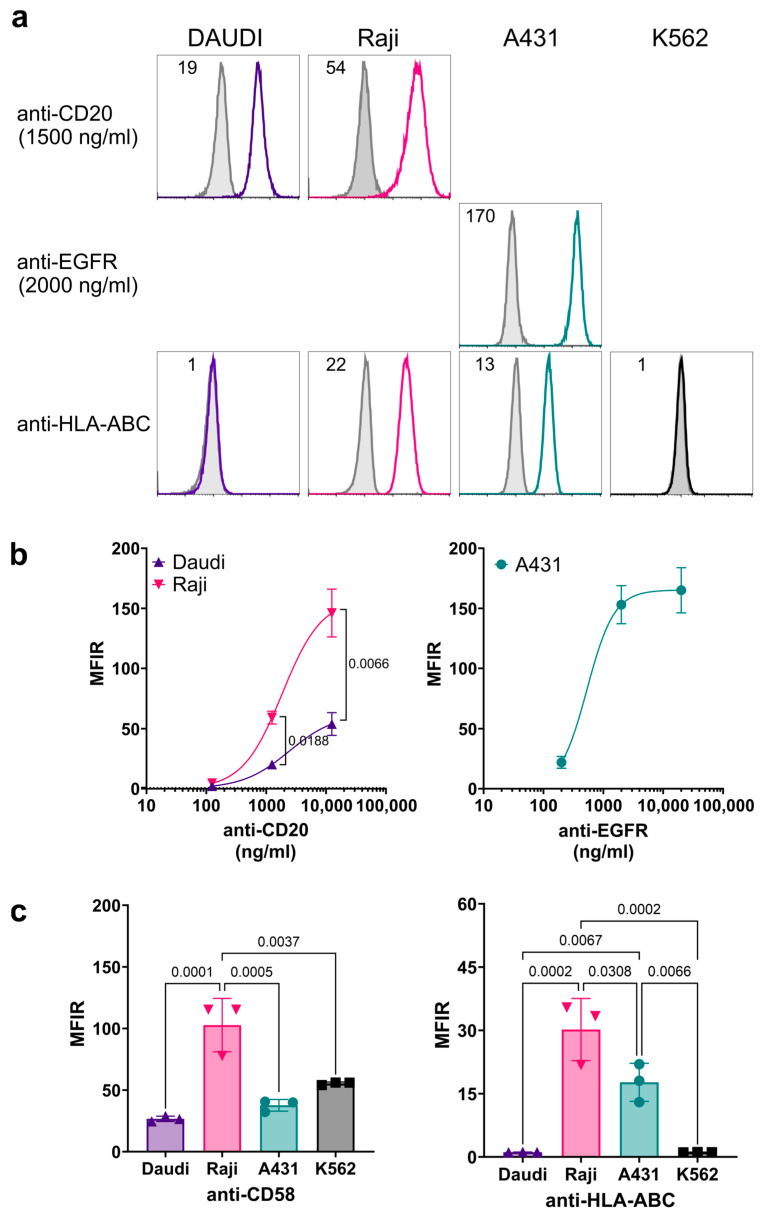
**Expression of surface antigen on target cells.** (**a**) Representative histogram overlays for the expressions of CD20, EGFR, and MHC-I (HLA-ABC), conducted by indirect and direct antibody staining using the humanized anti-CD20 and -EGFR at 1.25 and 2 µg/mL, respectively, and the mouse anti-HLA-ABC at a saturation condition. The antigen expression in each cell line is indicated next to the histogram, and the mean intensity ratio (MFIR) for each histogram is described in the upper-left corner. Gray histograms correspond to isotype controls. (**b**) Pooled data (n = 3) of MFIRs with respect to target cell lines at three different primary antibody concentrations for anti-CD20 and -EGFR. (**c**) Pooled data (n = 3) for HLA-ABC and CD58 expressions in target cells used in the study. Each symbol represents an independent experiment.

The target cells used to study cytotoxicity were characterized to avoid the misinterpretation of the results due to different levels of antigen expression. Figure 4a shows that the level of CD20 expression on Raji cells was higher than on Daudi cells, with MFIR values at 1250 ng/mL of 59.2 ± 5.4 and 20.2 ± 2.3, respectively (*p* = 0.0003, unpaired *t*-test). On the other hand, EGFR expression on A431 cells, as measured using cetuximab staining, reached saturation at 2000 ng/mL, with MFIR values of 153.0 ± 15.9 and 165.1 ± 18.8 at 2000 ng/mL and 20 μg/mL, respectively (Figure 4b). Additionally, the target cells were tested for two antigens that may affect NK cytotoxicity, HLA-ABC and CD58. We confirmed that the prototypic cell line K562 used in direct NK-92 cytotoxicity assays, as well as Daudi cells, lacked HLA-ABC expression (MFIRs of 1.09 ± 0.04 and 1.13 ± 0.04, respectively). In contrast, Raji and A431 expressed high (MFIR 30.2 ± 7.4) and moderate (MFIR 17.7 ± 4.5) HLA-ABC levels, respectively (Figure 4c, left). Finally, CD58, which is the ligand for the co-stimulatory receptor CD2 on NK cells, was expressed at higher levels on Raji compared to the other cell lines with MFIRs of 55.4 ± 1.1, 26.7 ± 2.2, 102.7 ± 21.7, and 37.6 ± 4.7 for K562, Daudi, Raji, and A431, respectively (Figure 4c, right).

### 3.4. FcγRIIIa/CD16-Transfected NK-92 Cell Lines Induce Antibody-Dependent Cell-Mediated Cytotoxicity (ADCC)

**Figure 5:**
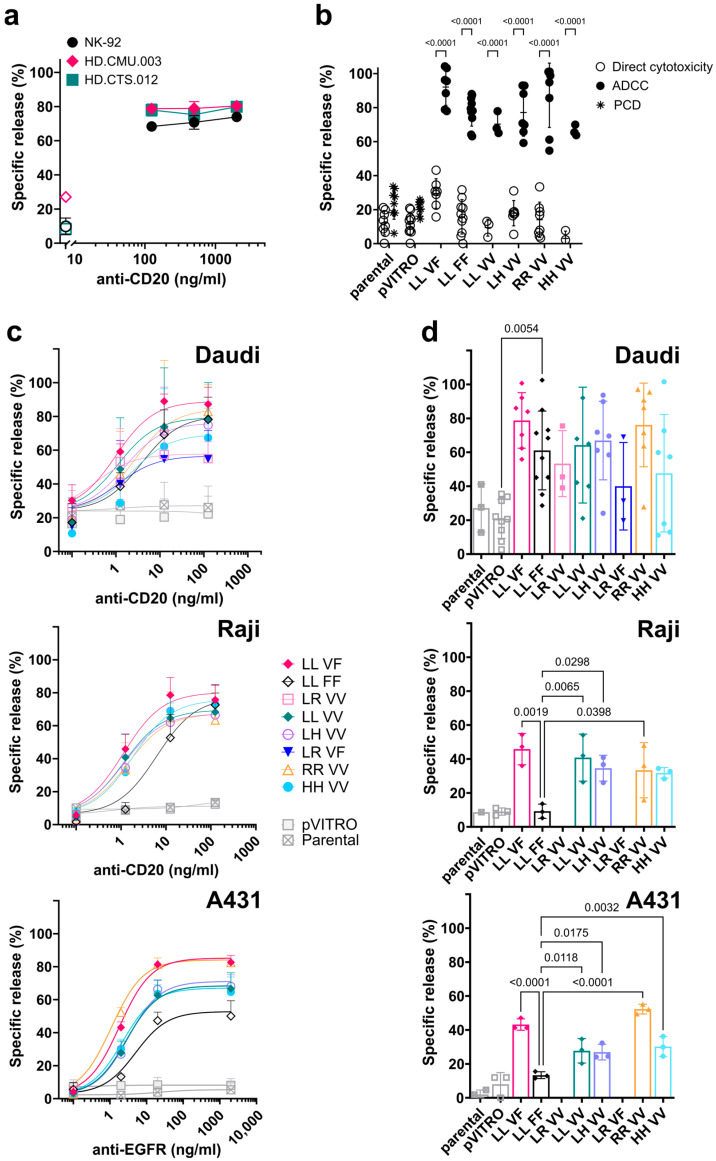
All NK-92 transfectants expressing FcγRIIIa/CD16 variants perform ADCC against antibody-coated cells. ADCC assays were performed using either Daudi or Raji cells as targets opsonized with the anti-CD20 antibody at an effector-to-target ratio (E:T) of 5:1 or A431 cells with anti-EGFR antibody at an E:T ratio of 10:1. The non-radioactive cytotoxic assays lasted for one and two hours for anti-CD20 and anti-EGFR, respectively. The graphs show the percentage of specific release (%) mean ± SD versus anti-CD20 or anti-EGFR. (**a**) Comparison of ADCC between the NK-92 ^LL_VF^ transfectant cells (black circle), NK cells from healthy donors isolated from blood (HD.CMU.003, magenta diamond), and a buffy coat (HD.CTS.012, green square) used as effectors, and Daudi cells as targets opsonized with different concentrations of anti-CD20 antibody (125, 500, and 2000 ng/mL) at an E:T of 5:1 (one experiment per donor). Direct cytotoxicity controls (i.e., no antibody, open symbols) are included. (**b**) This panel compares the six NK-92 cell lines and NK-92 controls co-cultured with Daudi cells alone (direct cytotoxicity, white circle), together with 500 ng/mL of anti-CD20 (ADCC, black circles), or NK-92 controls (parental and pVITRO) with targets and anti-CD20 (programmed cell death, PCD, stars). (**c**) The summary plots display the mean ± SD of the specific release (%) for ADCC by different transfected NK-92 cell lines, shown for Daudi (top, n = 5), Raji (middle, n = 3), and A431 (bottom, n = 3). The ADCC dose-response curves were fitted using the dose-response curves for the agonist model, where the agonist was the mAb, anti-CD20 or -EGFR, and the EC_50_ was estimated accordingly. (**d**) ADCC was measured at non-saturating concentrations of anti-CD20 and -EGFR mAbs, corresponding to 1.25 ng/mL and 2 ng/mL, respectively. The targets used were Daudi (n = 3 to 10, upper), Raji (n = 3, middle), and A431 (n = 3, bottom). Data were analyzed using one-way ANOVA, and multiple comparisons were made against NK-92 ^LL_FF^.

The parental NK-92 cell line has been extensively used to dissect the mechanisms of direct NK cytotoxicity. However, the lack of FcγRIIIa/CD16 expression makes them unsuitable for ADCC studies. The generation of a panel of NK-92 cell lines expressing different combinations of *FCGR3A* SNP variants, rs10127939 (L66H/R) and rs396991 (V176F), allowed us to test for differences in ADCC depending on a given SNP combination. First, one of the most common *FCGR3A* genotypes in the population, i.e., homozygosity for L and F, was analyzed. ADCC mediated by a transfected NK-92 ^LL_FF^ cell line against Daudi target cells was induced by an anti-CD20 mAb as compared to the ADCC induced by matching-genotype NK cells isolated from either fresh blood or buffy coat. As little as 125 ng/mL of anti-CD20 induced the maximal lysis of Daudi cells (~80%, Figure 5a) with no apparent differences between NK-92 ^LL_FF^ and primary NK cells despite the fact that primary NK cells have higher levels of FcγRIIIa/CD16 on their surface (Figure 1b,c).

ADCC was demonstrated for all NK-92 cell lines transfected with the allelic variants of FcγRIIIa/CD16, as shown in the summary plots (Figure 5b,c). The direct cytotoxicity of NK-92 transfectants against Daudi cells in the absence of anti-CD20, a control included in all experiments, was significantly lower than ADCC, with minor variations within the six NK-92 transfectants (Figure 5b, open circles). The FcγRIIIa/CD16 deficient controls, parental NK-92 and empty vector NK-92 ^pVITRO^ transfectant, induced direct cytotoxicity of the Daudi target cells with 12.2 ± 7.2% and 11.7 ± 6.4% of specific lysis, respectively. The addition of 500 ng/mL anti-CD20 antibody to the NK-92 control cells increased specific lysis by 11% compared to direct cytotoxicity (23.5 ± 9.2% and 21.5 ± 4.0%, respectively; Figure 5b, star symbols). Since the only difference between the two conditions was the presence of the anti-CD20 antibody, this additional killing was related to the direct induction of programmed cell death, a mechanism of target cell destruction that can be observed with type-II anti-CD20 antibodies such as Obinutuzumab [58,59,60]. Importantly, all eight NK-92 transfectants induced ADCC, significantly different from parental NK-92 and the NK-92 ^pVITRO^ cell lines, using Daudi cells as target (*p* ≤ 0.0001).

Overall, ADCC mediated by the transfected NK-92 cell lines against both CD20-expressing target cells was antibody dose-dependent when assayed at a fixed E:T ratio of 5:1 (Figure 5c: top Daudi; middle Raji). Maximum lysis of Daudi cells was achieved at 12.5 ng/mL of anti-CD20, with 56.3% and 55.9% specific lysis for NK-92 ^LR_VV^ and NK-92 ^LR_VF^, respectively, compared to an average of 79.4 ± 6.6% specific lysis for the other FcγRIIIa/CD16-expressing NK-92 transfectants. Of note, the low-affinity transfectant NK-92 ^LL_FF^ showed 79.1% specific lysis. In terms of the EC_50_, NK-92 ^LL_FF^ and NK-92 ^HH_VV^ had the highest (2.13 and 2.23 ng/mL), while NK-92 ^LR_VV^ had the lowest (0.02 ng/mL) value; all other transfectants had an EC_50_ ranging from 0.62 to 1.64 ng/mL. Nevertheless, using Raji target cells, which express more than twice as much CD20 as Daudi cells (Figure 4), the average maximum specific lysis (72.6 ± 5.6%) for the six NK-92 transfectants tested was observed at an anti-CD20 mAb concentration of 125 ng/mL. ADCC against Raji target cells mediated by NK-92 ^LL_FF^ showed an EC_50_ of 6.22 ng/mL in contrast to the average of 1.1 ng/mL observed for the other five NK-92 transfectants.

Finally, ADCC against the A431, a cell line expressing the EGFR antigen, was demonstrated for all NK-92 transfectants, with a maximum of lysis reached at 20 ng/mL. In the presence of an anti-EGFR mAb, the variants NK-92 ^LL_VF^ and NK-92 ^RR_VV^ showed the highest ADCC (85.3% and 84.1% specific lysis, respectively), followed by NK-92 ^LL_VV^, NK-92 ^LH_VV^, and NK-92 ^HH_VV^ (68.5, 71.1, and 67.1%, respectively), whereas the low-affinity variant NK-92 ^LL_FF^ showed only 52.8% of the maximum specific lysis (Figure 5c, bottom) and the highest EC_50_ (5.02 ng/mL, Table 1).

To better describe the differences between cell lines, particularly the NK-92 ^LL_FF^ variant, we analyzed the specific release of each target cell line at non-saturating concentrations of mAbs, which were 1.25 ng/mL and 2 ng/mL for anti-CD20 and -EGFR, respectively. The comparisons showed statistical differences in ADCC mediated by NK-92 ^LL_FF^ when the Raji and A421 cells were used as targets but not when Daudi cells were used (Figure 5d).

Finally, we tested whether there was a correlation between ADCC and FcγRIIIa/CD16 surface expression as detected using the 3G8 clone in overnight IL2-deprived NK-92 transfectants. No correlation was found for any of the three different target cell lines with R squared values of 0.1564, 0.2671, and 0.2110 and a two-tailed *p* of 0.4377, 0.938, and 0.3594 for Daudi, Raji, and A431 cells, respectively (Figure S5). 

In conclusion, all NK-92 cells transfected with different combinations of SNPs for *FCGR3A*, rs10127939 (L66H/R) and rs396991 (V176F), mediated ADCC, which could be clearly distinguished from direct cytotoxicity. 

There were minor differences in the lytic activity induced by anti-CD20 antibody binding to FcγRIIIa/CD16, independent of the genetic variant involved. Nevertheless, more pronounced differences were observed for anti-EGFR and against CD20-positive Raji target cells at non-saturating concentrations of anti-CD20 mAb. For example, ADCC against EGFR-positive A431 target cells in the presence of cetuximab was lower with the low-affinity variant expressed on NK-92 ^LL_FF^ compared to the high-affinity variants.

### 3.5. Direct Cytotoxicity against K562 Target Cells Mediated by FcγRIIIa/CD16-Transfected NK-92 Cell Lines

**Figure 6:**
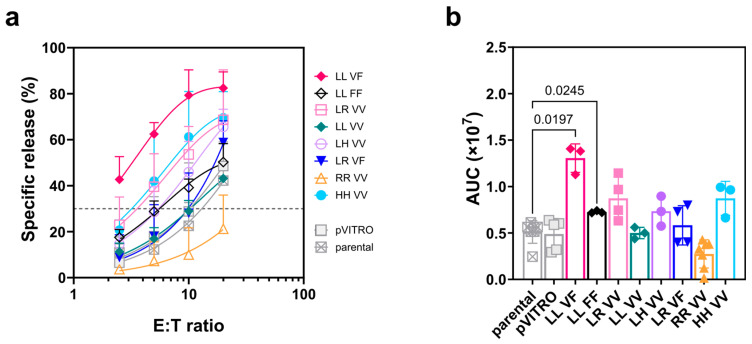
Direct cytotoxicity against K562 target cells by NK-92 transfectants expressing FcγRIIIa/CD16 variants. The direct cytotoxicity assay used K562 cells as the target. The cytotoxicity assay quantified the release of a non-radioactive compound resulting from the lysis/destruction of target cells over a two-hour period. NK-92 transfectants were cultured with 200 IU/mL of IL2 for two days and challenged with K562 at different effector-to-target (E:T) ratios. (**a**) Summary plots of specific release (%) are shown as the specific release mean ± SD of four independent experiments for each target cell line. Lytic units at 30% specific lysis (LU_30_) for 10^7^ effector cells were obtained by fitting the plot of pooled data using an asymptote-modified exponential growth model. (**b**) This is a summary plot that compares the mean ± SD of the area under the curve (AUC) calculated from individual cytotoxicity assays for the eight transfectants, including the parental and NK-92 ^pVITRO^ control cell lines. A comparison was made between the mean of NK-92 parental and the remaining NK-92 transfectants using Dunnett’s T3 multiple comparisons test. Values are not shown if *p* ≥ 0.05.

Direct cytotoxicity of IL2-deprived NK-92 transfectants against Daudi target cells showed little variation among the six cell lines tested (open circles, Figure 5b). To further test the direct NK cytotoxicity mediated by the FcγRIIIa/CD16-expressing NK-92 transfectants, we performed cytotoxicity assays against the prototypic HLA-ABC-negative K562 target cells in the presence of 200 IU/mL of IL2 (Figure 6). Several transfected NK-92 lines showed increased direct cytotoxicity compared to the FcγRIIIa/CD16-negative NK-92 control effector cells, parental NK-92, and NK-92 ^pVITRO^ lines (Figure 6a). To show all the pooled data, we plotted the results for all the different cell lines as described in the Materials section to obtain the LU_30_, i.e., the number of effector cells required to kill 30% of the K562 expressed per 10^7^ effectors (Table 2). Thus, the NK-92 ^LL_VV^ and NK-92 ^LR VF^ transfectants showed the same LU_30_ values as the control cell lines (parental and pVITRO), followed by the NK-92 ^LL_FF^ and NK-92 ^LH_VV^ transfectants. NK-92 ^LR_VV^ and NK-92 ^HH_VV^ required three times less effectors than controls to lyse 30% of the targets. Finally, the NK-92 ^LL_VF^ transfectant showed enhanced direct cytotoxicity (Figure 6a).

For the two most common *FCGR3A* allelic variant combinations in the European population, NK-92 ^LL_VF^ and NK-92 ^LL_FF^, the increase in direct NK cytotoxicity, measured as the area under the curve (AUC), reached statistical significance, with *p*-values of 0.0197 and 0.0245, respectively. In contrast, the NK-92 ^RR_VV^ transfectant showed a slightly lower cytotoxicity compared to the controls, which did not reach statistical significance (Figure 6b).

Finally, direct cytotoxicity mediated by IL2-deprived NK-92 transfectants against all four target cell lines was analyzed. The absence of HLA-ABC in K562 and Daudi, as compared to Raji and A431, was associated with higher direct cytotoxicity, when tested at an E:T ratio of 5:1, with specific lysis of 12.6 ± 12.8% (n = 11), 21.9 ± 7.8% (n = 11), 5.1 ± 2.7% (n = 8), and 4.1 ± 1.4% (n = 8), respectively, and with small differences between the different NK-92 transfectants. In contrast, no differences were observed against target cells expressing HLA-ABC (Figure S6).

In summary, this study demonstrated that all NK-92 cells transfected with different combinations of SNP for *FCGR3A* can effectively mediate ADCC, which is distinct from direct cytotoxicity. The lytic activity observed in ADCC varied depending on the genetic variant and the type of target cells. The expression of FcγRIIIa/CD16 resulted in an increase in direct cytotoxicity on some NK-92 transfectants as compared to parental NK-92 cells. These findings open new avenues of investigation.

## 4. Discussion

Immortal cell lines derived from leukemia or lymphoma are powerful tools for the study of NK cell biology (44). The NK-92 cell line was first reported by Klingemann and colleagues in 1994 [43,44,45]. This cell line lacks FcγRIIIa/CD16 surface expression for largely unexplained reasons and, therefore, does not perform ADCC. In recent years, attempts have been made to generate NK cell lines expressing FcγRIIIa/CD16 to study ADCC and for adoptive cancer immunotherapy using tumor antigen-directed mAbs [61]. Several publications describe the generation of NK-92 cell lines expressing FcγRIIIa/CD16 by viral transduction [48,49] or electroporation [47,62,63]. More recently, the modification of the promoter region of *FCGR3A* in the parental NK-92 cell line using CRISPR genome editing technology resulted in the endogenous expression of FcγRIIIa/CD16 [46]. Other attempts include tools to test the ADCC capacity of murine monoclonal antibodies using NK-92 cells transduced with murine CD16 [47]. Moreover, CAR-modified NK-92 cell lines against various tumor types and an NK-92 cell line expressing the high-affinity 176V variant of *FCGR3A*, also called high-affinity NK (haNK), have been reported. A robust antitumor activity in vitro and in vivo has been demonstrated using irradiated haNK cells in combination with IgG1 anti-tumor mAbs [64,65,66]. 

The focus of all these studies was on the SNP rs396991 (V176F), and to our knowledge, only Rataj et al. evaluated the impacts of both *FCGR3A* SNPs on ADCC. Nonetheless, the latter study analyzed the effect of FcγRIIIa/CD16 expression on T-cell function by transducing human T cells with three homozygous combinations of *FCGR3A* SNPs (HH_VV, LL_VV, and LL_FF) [67]. NK-92 cells were not tested in the latter study. A correlation between the two SNPs has been reported in European and Colombian populations [15,68]. This finding in European and Colombian populations was confirmed using an ‘LDpair Tool’ analysis [https://ldlink.nih.gov/, accessed on 27 July 2023], which only reports the L and H alleles for the tri-allelic variant rs10127939 (L66H/LR) without considering the R allele Table S6. In contrast, a linkage disequilibrium for rs10127939 and rs396991 was not found when ‘all populations’ (5008 individuals) were tested [69]. 

The present study is the first to successfully generate and characterize eight different NK-92 cell lines carrying these two most studied SNPs of *FCGR3A*. Despite the fact that there are 18 theoretical combinations, our panel of eight transfected NK-92 cell lines represents all combinations of the rs396991 (V176F) and rs10127939 (L66H/R) SNPs present in a human cohort of 87 European individuals, according to the study of Koene et al. The two most common combinations were LL_FF (32.2%) and LL_VF (39.1%) [15].

### 4.1. Expression of FcγRIIIa/CD16

The surface expression of the FcγRIIIa/CD16 depends on the association with signaling adaptor molecules such as TCRζ-chain (CD247) and/or FcγR1 [70]. Since Maki et al. demonstrated that NK-92 expresses CD247 at the mRNA level [45], we did not consider co-transfecting adaptor molecules to our NK-92 transfectants.

The analysis of FcγRIIIa/CD16 protein expression using different anti-CD16 mAb clones demonstrated levels within the same order of magnitude for all NK-92 transfectants. Moreover, FcγRIIIa/CD16 expression was higher compared to the previously reported transduced NK-92 cells [49] and, most importantly, was stable over time. In general, the expression levels of FcγRIIIa/CD16 on normal human NK cells were one to two orders of magnitude higher than on our NK-92 transfectants. Possible explanations for this finding include the (i) weaker activity of the plasmid promoter compared to the endogenous promoter, (ii) the downregulation of FcγRIIIa/CD16 in highly activated NK-92 cells due to the presence of high concentrations of IL2 in the culture media, (iii) the shedding of FcγRIIIa/CD16 by ADAM-17 [13,14], and (iv) elevated levels of miR218 compared to blood NK cells [71]. Indeed, we showed that overnight IL2 deprivation doubled the surface expression of FcγRIIIa/CD16 in the transfected NK-92 cell lines. However, without further experiments, we cannot draw firm conclusions to explain the overall lower FcγRIIIa/CD16 expression levels observed in the transfected NK-92 cell lines.

In our hands, the clone MEM154 discriminated poorly between V and F for the V176F SNP in transfectants, contrary to what we and others have previously described in NK cells isolated from peripheral blood [15] probably due to the lower expression of FcγRIIIa/CD16 in the transfected NK-92 cell lines. Indeed, the MEM154 signal was the weakest of all antibody clones tested. A problem with the reagent was excluded because MEM154 easily identified the SNP rs396991 (V176F) in primary NK cells. On the other hand, we are aware that clone 3G8 does not bind equally to F and V allotypes (rs396991), showing stronger binding to V, which is consistent with our flow cytometry results. Unfortunately, we did not have access to the reagent reported by Li et al. that binds equally to F and V allotypes [72]. In conclusion, our results indicate differences in the overall FcγRIIIa/CD16 expression levels on the NK-92 transfectants as compared to primary NK cells.

### 4.2. ADCC Mediated by NK-92 Expressing FcγRIIIa/CD16

Regardless of the difference in the expression level between the NK-92 transfectants and primary NK cells carrying the same SNP combination (LL_FF), ADCC was equivalent for CD20-expressing target cells. This indicates that the FcγRIIIa/CD16 expression levels on the NK-92 transfectants are sufficient for ADCC under our experimental conditions. Previously, it was reported that the combination of rs396991 (V176F) and rs10127939 (L66H/R) affected the binding of IgG, dividing the individuals in the study population into high (66RR or HH with 176VF or 176VV), intermediate (66LH or LR with 176VF or 176VV), and low binders (66LL with 176VF or 176FF; 66RR or 66HH with 176FF; 66LH with 176FF; 66RH with 176FF). However, ADCC assays were not performed in this study to analyze the effect of different SNPs combinations on NK cell function [73]. Interestingly, the IgG low binders, grouped by the combinations of rs396991 (V176F) and rs10127939 (L66H/R), conferred an increased risk for lupus nephritis in African Americans. The authors speculate that less efficient immune complex handling by low binders may contribute to lupus pathogenesis and that targeting FcγRIIIa/CD16 to improve receptor expression and function may be a potential therapeutic approach. To our knowledge, there are no other studies reporting the clinical relevance of different combinations of rs396991 and rs10127939 *FCGR3A* SNPs. However, rs396991 has been extensively studied and reported to be relevant in infection, autoimmunity, and inflammation [21,40,41,42].

Although IL2, added to maintain NK-92 cultures, has a major impact on the overall NK killing machinery as recently highlighted by transcriptomic analysis for activating and inhibitory receptors [74], ADCC mediated by NK-92 transfectants was clearly depended on IgG1 monoclonal antibody concentrations, as demonstrated by the dose-response curves. No major differences were observed in the lytic activity against two different CD20-positive target cell lines at saturating antibody concentrations, regardless of the genetic variant tested. However, compared to NK-92 ^LL_VF^ and other variants, ADCC induced by the low-affinity variant NK-92 ^LL_FF^ was indeed lower against EGFR-positive A431 target cells in the presence of cetuximab and against CD20-positive Raji target cells at non-saturating concentrations of anti-CD20 mAb. In addition to the role of the two SNPs, rs396991 (V176F) and rs10127939 (L66H/R), other factors may be considered. Several other molecular interactions between the antigen, the mAb, and the FcγRIIIa/CD16 could be involved to explain these findings. These include (i) the antigen density on the target cells; (ii) the epitope recognized by the antibody paratope (which may induce or block conformational changes leading to oligo- or dimerization of the antigen); or (iii) the fluidity of the cell membrane (given by fatty acid composition and raft formation), which may favor or impede the binding of the Fc portion to the FcγRIIIa/CD16. For example, the anti-EGFR mAbs cetuximab and panitumumab both interact with the extracellular domain of EGFR. Bárta et al. found that the binding of these mAbs to the EGFR depends on the cell line used to measure the antibody affinity. The anti-EGFR binding affinity to the keratinocyte cell line HaCaT was higher and more heterogeneous as compared to A431. These differences, detected using interaction map analysis, suggest that it is not the target but the supramolecular context in which the antigen is located that determines the binding of the mAb [75]. Thus, the conflicting data stemming from therapeutic mAb studies could be the result of focusing only on the genetic variations in *FCGR3A* and not considering variations in the antigen levels on the targeted cells. 

### 4.3. Direct or Natural Cytotoxicity of NK-92 Transfectants

Unexpectedly, several FcγRIIIa/CD16-transfected NK-92 lines showed increased direct cytotoxicity against HLA-ABC class-negative K562 target cells compared to FcγRIIIa/CD16-negative NK-92 control cells. A role for FcγRIIIa/CD16 in direct cytotoxicity independent of antibody ligation has been reported, but the mechanisms remain elusive [76]. Target cells may express putative ligands for FcγRIIIa/CD16; alternatively, FcγRIIIa/CD16 may interact with other activating receptors expressed on NK cells. Supporting this hypothesis, physiological interactions between FcγRIIIa/CD16 and the co-stimulatory receptor CD2 were shown to be necessary to induce direct NK cytotoxicity but not ADCC in a patient with EBV-driven Castleman’s disease homozygous for H in position 66 (rs10127939) [19]. Interactions between CD58 and CD2 are essential for immune synapse formation and target cell elimination by NK cells [77,78]; however, the observed differences in ADCC and direct NK cytotoxicity could not be explained by differences in the expression levels of CD58 on our target cells. Furthermore, CD2 is expressed by NK-92 cells [43,44,45,77,78] and by our NK-92 transfectants, including the parental line and NK-92 ^pVITRO^ transfected NK-92 cells Figure S7).

The NK-92 ^RR_VV^ cell line showed lower direct cytotoxicity against K562 cells compared to NK-92 ^LL_VF^. Here, we could speculate that single or multiple random plasmid integrations into the genomic DNA led to a dysregulation of pathways involved in direct cytotoxicity [79]. It is worth mentioning that the parental NK-92 cell line has numerical and structural cytogenic alterations, making it difficult to predict which chromosomes these alterations will be inserted into [43]. At last, the NK-92 ^HH_VV^ transfectant showed similar direct NK cytotoxicity against K562 targets compared to the parental NK-92 line. This finding contrasts with some previous reports of patients homozygous for H at position 66 of FcγRIIIa/CD16 (rs10127939) with recurrent infections and deficient NK cytotoxicity [18,19,20]. However, our results are consistent with findings in a patient with DiGeorge syndrome, homozygous for H, and with normal NK cytotoxicity, leading the authors to conclude that the L66H variant is unlikely to be a direct genetic cause of NK cell dysfunction [80]. 

## 5. Conclusions

In summary, we have generated eight NK-92 transfectants carrying different combinations of two important SNPs of *FCGR3A*, covering all major genotypes described in the human population. The functional characterization of these cell lines revealed differences in ADCC and direct NK cytotoxicity that may have implications for immunotherapy. The use of either patient-derived NK cells or “off-the-shelf” NK-92 cells offers the potential to modify or include the *FCGR3A* SNP combination in a more effective manner, depending on the desired activity. For instance, the LL_VF SNP combination demonstrated potent ADCC and direct cytotoxicity. In addition, other mAb engineering approaches such as afucosylation alter the affinity of IgG to the FcγRIIIa/CD16. 

## Figures and Tables

**Table 1:** EC_50_ obtained from ADCC dose-dependent curves using different NK-92 cell lines against target cells.

	Daudi			Raji			A431		
Transfectant	n	* R^2^	‡ EC_50_ (ng/mL)	n	R^2^	EC_50_ (ng/mL)	n	R^2^	EC_50_ (ng/mL)
NK-92 ^LL_VF^	2, 5	0.9139	0.99	3	0.9268	1.1	3	0.9715	1.8
NK-92 ^LL_FF^	4, 8	0.8504	2.13	3	0.9039	6.2	3	0.9011	5.0
NK-92 ^LR_VV^	3	0.5515	0.02	ND			ND		
NK-92 ^LL_VV^	4	0.5578	1.02	3	0.8626	1.2	3	0.9284	2.8
NK-92 ^LH_VV^	2, 4	0.7898	1.58	3	0.9106	1.1	3	0.9472	3.1
NK-92 ^LR_VF^	3	0.5181	0.62	ND			ND		
NK-92 ^RR_VV^	2, 5	0.7526	1.64	3	0.9749	1.4	3	0.9689	1.1
NK-92 ^HH_VV^	2, 4	0.5679	2.23	3	0.8003	1.8	3	0.9379	2.2
Parental	3, 6	0.0476	-;	3		-	3	0.0097	-
NK-92 ^pVITRO^	7, 9	0.0738	-	3		-	3	0	-

* The goodness of fit (R^2^) for the curves was calculated. For R^2^ < 0.5, poor fitting to asymptotic curves. ‡ Estimate of EC_50_ for calculated from the fitted curves. *Abbreviations:* EC_50_, effective concentration to reach 50% of cytotoxicity; ND, not determined.

**Table 2:** Comparison of the direct cytotoxicity of NK-92 transfectants against K562 cells by using the lytic units at 30% specific lysis (LU_30_) for 10^7^ effector cells.

Name	Goodness of Fit, R^2^ *	Maximum Specific Lysis (%)	LU_30_/10^7^ Effectors
NK-92 ^LL_VF^	0.8095	83	63
NK-92 ^LL_FF^	0.8588	52	18
NK-92 ^LR_VV^	0.6181	71	28
NK-92 ^LL_VV^	0.9251	55	10
NK-92 ^LH_VV^	0.8174	80	18
NK-92 ^LR_VF^	0.7148	100 *	11
NK-92 ^RR_VV^	0.3276	77	n/a †
NK-92 ^HH_VV^	0.6209	73	31
parental	0.6521	91	8
NK-92 ^pVITRO^	0.5487	83	10

* The goodness of fit (R^2^) for the curves was calculated. For R^2^ < 0.5, poor fitting curves. Constriction, specific release < 100%. † n/a, not applicable, as the cells never reached 30% of specific lysis.

## Data Availability

The data presented in this study are available on request from the corresponding authors.

## Supplementary Materials

The following supporting information can be downloaded at: https://www.mdpi.com/article/10.3390/antib13030055/s1, Figure S1: Gating strategy used in flow cytometry analysis of NK-92 cell lines; Figure S2: GenScriptpUC19-Amp *FCGR3A* transcript variant 3; Figure S3: Sanger sequencing of the messenger RNA of *FCGR3A* coming from human NK cells; Figure S4: Clones B73.1 and MEM154 confirmed the findings of 3G8 for the increase in FcγRIIIa/CD16 due to the lack of interleukin-2 during overnight cultures; Figure S5: Correlation in NK-92 transfectants between the expression of FcγRIIIa/CD16 and antibody-dependent cell-mediated cytotoxicity (ADCC); Figure S6: Comparison of direct cytotoxicity between target cells expressing, or not, HLA-ABC; Figure S7: Expression of CD2 on NK-92 cell lines. Table S1: Detailed list of reagents, antibodies, culture media, supplements, and cell lines used; Table S2: List of primer pairs used for the generation of *FCGR3A* allelic variants for SNPs rs396991 and rs10127939, including verification steps; Table S3: Names of constructs and allelic variants used in the current study; Table S4: Nomenclature of NK-92 cell lines carrying, or not, FCGR3A allelic variants, and plasmid used for their generation; Table S5: Expression level of FcγRIIIa/CD16 on NK-92 cells and NK cells using three different monoclonal antibody clones. Table S6: Linkage disequilibrium (LD) analysis for rs10127939 and rs396991 using LDpair Tool.
