# Supplementary material for: NK Cytotoxicity Mediated by NK-92 Cell Lines Expressing Combinations of Two Allelic Variants for FCGR3
Source: Antibodies (Basel). 2024 Jul 12;13(3):55. doi: 10.3390/antib13030055 (PMC11270249; doi:10.3390/antib13030055)
Supplement: Supplementary file 1 [file antibodies-13-00055-s001.zip › antibodies-3001059-supplementary.pdf]

antibodies

MDPI

Appendix A

Supplementary Tables

Table S1. Detailed list of reagents, antibodies, culture media, supplements, and cell lines used.

Reagent	Abbreviation	Brand	Town, Country	Cat. N°	RRID:
**Biologicals**					
Cetuximab, IgG1	Anti-EGFR	Invivogen	San Diego, CA, USA	hegfr-mab1	AB_3064809
Obinutuzumab, non-glycoengineered	Anti-CD20	Gift	Zurich, Switzerland		
Proleukin (Human recombinant interleukin 2)	IL2	Novartis Pharma	Basel, Switzerland	Proleukin	
**Antibodies**					
**Reagent**	**Abbreviation**	**Brand**	**Town, Country**	**Cat. N°**	**RRID:**
Anti-CD16, clone 3G8	3G8	BioLegend	San Diego, CA, USA	302008	AB_314208
Anti-CD16, clone B73.1	B73.1	BioLegend	San Diego, CA, USA	360704	AB_2562749
Anti-CD16, clone MEM154	MEM154	Thermo Fisher Scientific	Waltham, MA, US	MA1-19563	AB_1071355
Anti-CD58, clone TS2/9	–	BioLegend	San Diego, CA, USA	330905	AB_1186063
Anti- HLA-ABC, clone W6/32	–	BioLegend	San Diego, CA, USA	311405	AB_314874
Mouse IgG1 isotype, clone MOPC-21	–	BioLegend	San Diego, CA, USA	400108	AB_326429
Mouse IgG1 isotype, clone MOPC-21	–	BioLegend	San Diego, CA, USA	400112	AB_2847829
Mouse IgG2a isotype, clone 20102	–	R&D Systems	Minneapolis, MN, US	IC003P	AB_357245
F(ab')2 Fragment Goat Anti-Human IgG, F(ab')2 Fragment	BCR blocking	Jackson ImmunoResearch Labs	West Grove, PA, US	109-006-097	AB_2337550
Goat F(ab’)2 anti-human κ	–	SouthernBiotech	Birmingham, AL, US	2063-09	AB_2795748
Goat F(ab’)2 isotype control	–	SouthernBiotech	Birmingham, AL, US	0110-09	AB_2793959
**Chemicals, culture media and supplements**					
**Reagent**	**Abbreviation**	**Brand**	**Town, Country**	**Cat. N°**	**RRID:**
4-(2-hydroxyethyl)-1-piperazineethanesulfonic acid	HEPES	Gibco	Grand Island, NY, US	42401-018	n/a
7-Aminoactinomycin D	7-AAD	BD Biosciences	San Jose, CA, US	559925	n/a
Bovine serum albumin	BSA	Sigma-Aldrich	St. Louis, MO, US	A2153	n/a

Dulbecco's Modified Eagle Medium	DMEM	Gibco	Grand Island, NY, US	41965039	n/a
Dulbecco's Phosphate-Buffered Saline 1×	PBS	Gibco	Grand Island, NY, US	14190-094	n/a
Ethylenediaminetetraacetic acid	EDTA	Sigma-Aldrich	St. Louis, MO, US	ED4S	n/a
Fetal bovine serum	FBS	Sigma-Aldrich	St. Louis, MO, US	F7524	n/a
Ficoll-Paque PLUS	–	GE Healthcare	Uppsala, Sweden	17-144-02	n/a
Geneticin (G-418 sulfate)	–	Gibco	Grand Island, NY, US	10131-027	n/a
L-Alanyl-L-Glutamine	L-Ala/L-Glu	Bioswisstec	Schaffhausen, Switzerland	K0302	n/a
Minimum Essential Medium Amino Acids Solution	EAA	Gibco	Grand Island, NY, US	11130051	n/a
Non-Essential Amino Acids Solution	NEAA	Gibco	Grand Island, NY, US	11140035	n/a
Penicillin/streptomycin 100×	Pen/Strep	Gibco	Grand Island, NY, US	15140-122	n/a
Probenecid	–	Sigma-Aldrich	St. Louis, MO, US	P8761	n/a
GelRed Nucleic Acid Gel Stain	–	Biotium Inc	Fremont, CA, US	41003	n/a
Roswell Park Memorial Institute 1640	RPMI	Gibco	Grand Island, NY, US	22400089	n/a
Sodium pyruvate	–	Gibco	Grand Island, NY, US	11360-039	n/a
**Cell lines**					
**Reagent**	**Abbreviation**	**Brand**	**Town, Country**	**Cat. N°**	**RRID:**
A-431	–	ATCC	Manassas, VA, US	CRL-1555	CVCL_0037
Daudi	–	ATCC	Manassas, VA, US	CCL-213	CVCL_0008
K562	–	ATCC	Manassas, VA, US	CCL-243	CVCL_2142
NK-92	–	ATCC	Manassas, VA, US	CRL-2407	CVCL_2142
noGFP-CD16 176F NK-92.05	–	Gift			
Raji	–	ATCC	Manassas, VA, US	CCL-86	CVCL_0004
**Molecular Biology**					
**Reagent**	**Abbreviation**	**Brand**	**Town, Country**	**Cat. N°**	**RRID:**
100bp ladder	–	Thermo Fisher Scientific	Waltham, MA, US	SM0321	n/a
AgeI	–	New England BioLabs	Ipswich, MA, US	R3552	n/a
Amaxa Cell Line Nucleofector® Kit R	–	Lonza	Stein, Switzerland	VCA-1001	n/a

Ampicillin	–	Sigma-Aldrich	St. Louis, MO, US	A8351	n/a
Deoxynucleotide Solution Mix	dNTP	New England Biolabs	Ipswich, MA, US	N0447	n/a
Instant Sticky-end Ligase master mix	–	New England BioLabs	Ipswich, MA, US	M0370S	n/a
Monarch PCR & DNA Cleanup Kit	–	New England BioLabs	Ipswich, MA, US	T1030	n/a
NEB 5-alpha Competent *E. coli*	–	New England BioLabs	Ipswich, MA, US	C2987	n/a
NheI	–	New England BioLabs	Ipswich, MA, US	R3131	n/a
ProtoScript II First Strand cDNA Synthesis Kit	–	New England Biolabs	Ipswich, MA, US	E0552S	n/a
Q5 high-fidelity DNA polymerase	–	New England BioLabs	Ipswich, MA, US	M0491	n/a
Q5 Site-Directed Mutagenesis kit	–	Promega	Madison, WI, US	E0552S	n/a
Qubit 1× dsDNA High Sensitivity (HS) kit	–	Invitrogen	Carlsbad, CA, US	Q33231	n/a
Random Primer Mix	–	New England Biolabs	Ipswich, MA, US	S1330A	n/a
TruSeq Nano DNA High Throughput Library Prep kit	–	Illumina	San Diego, CA, US	20015965	n/a
Tryzol Reagent	–	Invitrogen	Carlsbad, CA, US	15596018	n/a

Other commercial kits

Reagent	Abbreviation	Brand	Town, Country	Cat. N°	RRID:
DELFIA EuTDA Cytotoxicity Reagents	–	PerkinElmer	Waltham, MA, USA	AD0116	n/a
LS columns	–	Miltenyi Biotec	Cologne, Germany	130-042-401	n/a
NK isolation kit, human	–	Miltenyi Biotec	Cologne, Germany	130-092-657	n/a

Abbreviations. n/a, not applicable

Table S2. List of primers pairs used for the generation *FCGR3A* allelic variants for SNPs rs396991 and rs10127939, including verification steps

Name	Sequence (5′– to –3′)*	Purpose
MSC2 AgeI 16A F	CAT Gac cgg tGA TTC TGT GTG	Clone *FCGR3A* into *pVITRO-neo-mcs*, MCS2 using *AgeI* and *NheI* restriction enzymes
MSC2 NheI 16A R	TGC TTA Ggc tag cAT CTA GAG	Clone *FCGR3A* into *pVITRO-neo-mcs*, MCS2 using *AgeI* and *NheI* restriction enzymes
SDM CD16A 66L_H F	AAT GAG AGC CAC ATC TCA AGC	SDM T to A (L to H) of the rs10127939
SDM CD16A 66 R	GTG AAA CCA CTG TGT GGA ATT G	SDM T to A (L to H) of the rs10127939
SDM CD16A 66L_R F	AAT GAG AGC CGC ATC TCA AGC	SDM T to G (L to R) of the rs10127939

SDM CD16A 66 R	GTG AAA CCA CTG TGT GGA ATT G	SDM T to G (L to R) of the rs10127939
SDM F158_V Fw	CAG GGG GCT TTT TGG GAG TAA AAA T	SDM G to T (V to F) of the rs396991
SDM F158_V RV	CAG AAG TAG GAG CCG CTG	SDM G to T (V to F) of the rs396992
MCS2	TTT TGA GCG GAG CTA ATT CTC GGG	Sanger sequencing primers for constructs
Seq 48_158 fw	GCT CGA GAA GGA CAG TGT GA	cDNA amplification
Seq 48_158 rev	GGT TGA CAC TGC CAA ACC TT	cDNA amplification

* In lowercase nucleotides, restriction enzyme sites; underlined the single nucleotide change for SDM where in bold is highlighted the codon corresponding to the amino acid of relevance.

Table S3. Name of constructs and allelic variants used in the current study

Plasmid Name	Source
pUC19-CD16A v3	*GenScript pUC19-Amp* and *FCGR3A transcript variant 3. (NM_001127593.1). L and V for rs10127939 and rs396991*
pVITRO-neo-mcs	*InvivoGen* (Cat# pvitro1-nmcs)
pVITRO-neo-CD16A_VL$_{MCS2}$ *b*	Cloning of *pUC19-CD16A v3* into *pVITRO-neo-mcs*
pVITRO-neo-CD16A_VH$_{MCS2}$ a	SDM of *pVITRO-neo-CD16A_VL*$_{MCS2}$ *b*
pVITRO-neo-CD16A_VR$_{MCS2}$ *a*	SDM of *pVITRO-neo-CD16A_VL*$_{MCS2}$ *b*
pVITRO-neo-CD16A_FL$_{MCS2}$ *4*	SDM of *pVITRO-neo-CD16A_VL*$_{MCS2}$ *b*
pVITRO-neo-CD16A_FL$_{MCS2}$ *13*	SDM of *pVITRO-neo-CD16A_VL*$_{MCS2}$ *b*

Table S4. Nomenclature of NK-92 cell lines carrying or not *FCGR3A* allelic variants and plasmid used for their generation.

Name	Freq. (%)	Plasmid's name
Parental NK-92	n/a	n/a
NK-92 ^{pVITRO}	n/a	*pVITRO-neo-mcs*
NK-92 $^{LL\_VF}$	39.1	*pVITRO-neo-CD16A_VL*$_{MCS2}$ *b* and *pVITRO-neo-CD16A_FL*$_{MCS2}$ *4*
NK-92 $^{LL\_FF}$	32.2	*pVITRO-neo-CD16A_FL*$_{MCS2}$ *4*
NK-92 $^{LH\_VF}$	6.9	not done
NK-92 $^{LR\_VV}$	6.9	*pVITRO-neo-CD16A_VL*$_{MCS2}$ *b* and *pVITRO-neo-CD16A_VR*$_{MCS2}$ *a*
NK-92 $^{LL\_VV}$	4.6	*pVITRO-neo-CD16A_VL*$_{MCS2}$ *b*
NK-92 $^{LH\_VV}$	4.6	*pVITRO-neo-CD16A_VL*$_{MCS2}$ *b* and *pVITRO-neo-CD16A_VH*$_{MCS2}$ *a*
NK-92 $^{LR\_VF}$	4.6	*pVITRO-neo-CD16A_VR*$_{MCS2}$ *a* and *pVITRO-neo-CD16A_FL*$_{MCS2}$ *13*
NK-92 $^{RR\_VV}$	1.1	*pVITRO-neo-CD16A_VR*$_{MCS2}$ *b*
NK-92 $^{HH\_VV}$	<1	*pVITRO-neo-CD16A_VH*$_{MCS2}$ *a*

* Estimated frequency in a population of 87 healthy donors obtained from Koene et al [15].

15 Koene, H. R., M. Kleijer, J. Algra, D. Roos, A. E. von dem Borne, and M. de Haas. "Fc Gammariiia-158v/F Polymorphism Influences the Binding of Igg by Natural Killer Cell Fc Gammariiia, Independently of the Fc Gammariiia-48l/R/H Phenotype." *Blood* 90, no. 3 (1997): 1109-14.

Table S5. Expression level of FcγRIIIa/CD16 on NK-92 cells and NK cells using three different monoclonal antibody clones

	B73.1		3G8		MEM154	
**Cells**	**MFIR ± SD**	**n**	**MFIR ± SD**	**n**	**MFIR ± SD**	**n**
NK-92 $^{LL\_VF}$	14.3 ± 7.9	13	50.7 ± 26.6	15	2.2 ± 0.6	15
NK-92 $^{LL\_FF}$	27.1 ± 14.0	10	42.5 ± 23.2	12	1.0 ± 0.0	12
NK-92 $^{LR\_VV}$	5.9 ± 5.5	2	23.8 ± 22.2	2	1.7 ± 0.6	2
NK-92 $^{LL\_VV}$	25.4 ± 9.0	10	91.9 ± 38.4	12	5.5 ± 1.2	12
NK-92 $^{LH\_VV}$	7.9 ± 1.9	10	42.6 ± 17.13	12	3.2 ± 0.7	12
NK-92 $^{LR\_VF}$	3.2 ± 1.6	5	13.0 ± 4.2	6	1.4 ± 0.3	6
NK-92 $^{RR\_VV}$	3.5 ± 1.4	10	63.7 ± 32.9	12	4.6 ± 1.1	12
NK-92 $^{HH\_VV}$	0.8 ± 0.1	10	41.7 ± 18.6	12	2.8 ± 0.6	12
parental NK-92	0.7 ± 0.2	12	0.9 ± 0.1	9	1.0 ± 0.0	12
NK-92 pVITRO	0.9 ± 0.2	15	0.9 ± 0.1	15	1.0 ± 0.0	15
noGFP-CD16 176F NK-92.05	1.9 ± 1.1	3	6.1 ± 2.2	4	1.1 ± 0.0	4
Human NK LL_VF	1459	1	206	1	43	1
Human NK LL_FF	1397	1	80	1	1.0	1

Table S6. Linkage disequilibrium (LD) analysis for rs10127939 and rs396991 using LDpair Tool. We used the "LDpair Tool" extracting data from the 1000 Genomes Project (GRCh38 High Coverage annotation) to investigate the correlation of alleles for a pair of variants in high linkage disequilibrium (LD) [69].

	European	Colombian	All populations
**N**	1,006	188	5,008
**D′**	0.9615	1.0	0.9774
**R^2**	0.1453	0.1649	0.0518
**X^2**	146.1551	30.9967	259.2509
***p*-value**	< 0.0001	< 0.0001	< 0.0001
**Interpretation***	rs10127939 (A→L) allele is **correlated** with rs396991 (A→F) allele<br>rs10127939 (T→H) allele is **correlated** with rs396991 (C→V) allele	rs10127939 (A→L) allele is **correlated** with rs396991 (A→F) allele<br>rs10127939 (T→H) allele is **correlated** with rs396991(C→V) allele	rs10127939 and rs396991 are in **linkage equilibrium**

* Correlated Alleles. Alleles that are correlated if linkage disequilibrium is present ($R^2 > 0.1$). If linkage equilibrium, no alleles are reported.

69 Machiela, M.J.; Chanock, S.J. LDlink: A Web-Based Application for Exploring Population-Specific Haplotype Structure and Linking Correlated Alleles of Possible Functional Variants. *Bioinformatics* **2015**, *31*, 3555–3557. https://doi.org/10.1093/bioinformatics/btv402.

Supplementary Figure Legends

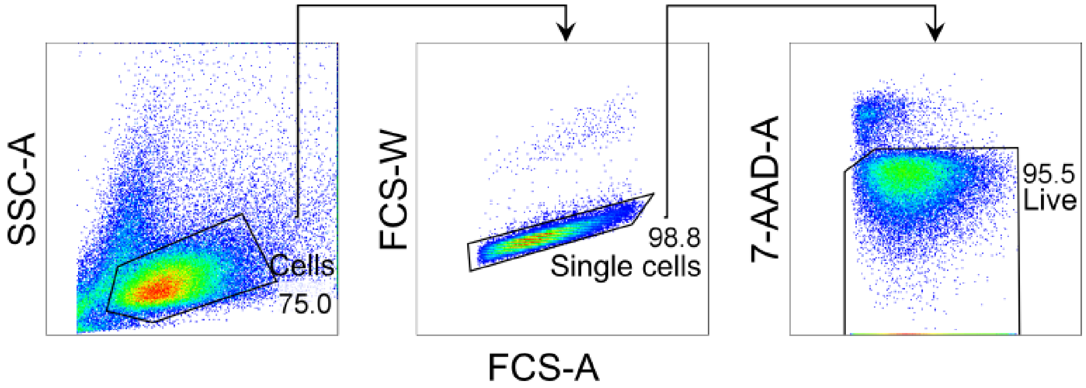

Figure S1. Gating strategy used in flow cytometry analysis of NK-92 cell lines.

For the flow cytometry analysis of NK-92 transfectants, cells were first gated based on their size and granularity to exclude cell debris (left). Next, cell aggregates were excluded, and a selection of single cell population was selected (middle). Finally, only the live cells were chosen using the dead cell exclusion dye 7-AAD (7-amino-actinomycin D). The figures display pseudo-color plots for forward scatter-area (FSC-A) versus side scatter-area (SSC-A), forward scatter-width (FCS-W), and 7-AAD-area (7-AAD-A). The names of gates and percentage of gated events are shown in each plot.

```
ATG TGG CAG CTG CTC CTC CCA ACT GCT CTG CTA CTT CTA GTT TCA GCT GGC ATG CGG ACT GAA GAT CTC CCA AAG GCT GTG GTG TTC CTG GAG CCT CAA T  < 100
M   W   Q   L   L   L   P   T   A   L   L   L   L   V   S   A   G   M   R   T   E   D   L   P   K   A   V   V   F   L   E   P   Q   W
            10           20            30            40           50            60           70           80            90

                                                                                                                                 >L48H/R
                                                                                                                                 |
GG TAC AGG GTG CTC GAG AAG GAC AGT GTG ACT CTG AAG TGC CAG GGA GCC TAC TCC CCT GAG GAC AAT TCC ACA CAG TGG TTT CAC AAT GAG AGC CTC AT  < 200
   Y   R   V   L   E   K   D   S   V   T   L   K   C   Q   G   A   Y   S   P   E   D   N   S   T   Q   W   F   H   N   E   S   L   I
           110          120           130          140           150           160          170          180           190

C TCA AGC CAG GCC TCG AGC TAC TTC ATT GAC GCT GCC ACA GTC GAC GAC AGT GGA GAG TAC AGG TGC CAG ACA AAC CTC TCC ACC CTC AGT GAC CCG GTG  < 300
  S   S   Q   A   S   S   Y   F   I   D   A   A   T   V   D   D   S   G   E   Y   R   C   Q   T   N   L   S   T   L   S   D   P   V
            210          220           230          240           250          260           270           280          290

CAG CTA GAA GTC CAT ATC GGC TGG CTG TTG CTC CAG GCC CCT CGG TGG GTG TTC AAG GAG GAA GAC CCT ATT CAC CTG AGG TGT CAC AGC TGG AAG AAC A  < 400
Q   L   E   V   H   I   G   W   L   L   L   Q   A   P   R   W   V   F   K   E   E   D   P   I   H   L   R   C   H   S   W   K   N   T
           310          320          330           340          350          360            370          380          390

CT GCT CTG CAT AAG GTC ACA TAT TTA CAG AAT GGC AAA GGC AGG AAG TAT TTT CAT CAT AAT TCT GAC TTC TAC ATT CCA AAA GCC ACA CTC AAA GAC AG  < 500
   A   L   H   K   V   T   Y   L   Q   N   G   K   G   R   K   Y   F   H   H   N   S   D   F   Y   I   P   K   A   T   L   K   D   S
           410          420           430          440           450           460          470          480           490

                                        >V158F
                                        |
C GGC TCC TAC TTC TGC AGG GGG CTT TTT GGG AGT AAA AAT GTG TCT TCA GAG ACT GTG AAC ATC ACC ATC ACT CAA GGT TTG GCA GTG TCA ACC ATC TCA  < 600
  G   S   Y   F   C   R   G   L   F   G   S   K   N   V   S   S   E   T   V   N   I   T   I   T   Q   G   L   A   V   S   T   I   S
            510          520           530          540           550          560           570           580          590

TCA TTC TTT CCA CCT GGG TAC CAA GTC TCT TTC TGC TTG GTG ATG GTA CTC CTT TTT GCA GTG GAC ACA GGA CTA TAT TTC TCT GTG AAG ACA AAC ATT C  < 700
S   F   F   P   P   G   Y   Q   V   S   F   C   L   V   M   V   L   L   F   A   V   D   T   G   L   Y   F   S   V   K   T   N   I   R
           610          620          630           640          650          660           670          680          690

GA AGC TCA ACA AGA GAC TGG AAG GAC CAT AAA TTT AAA TGG AGA AAG G  < 748
   S   S   T   R   D   W   K   D   H   K   F   K   W   R   K   X
           710          720           730          740
```

Figure S2. GenScriptpUC19-Amp *FCGR3A* transcript variant 3.

The commercial plasmid's *FCGR3A* sequence was verified by Sanger sequencing. The nucleotide and translated amino acid sequences were generated using SerialCloner 2.6.1. The rs10127939 (L66H/R) and rs396991 (V176F) variants are highlighted in red.

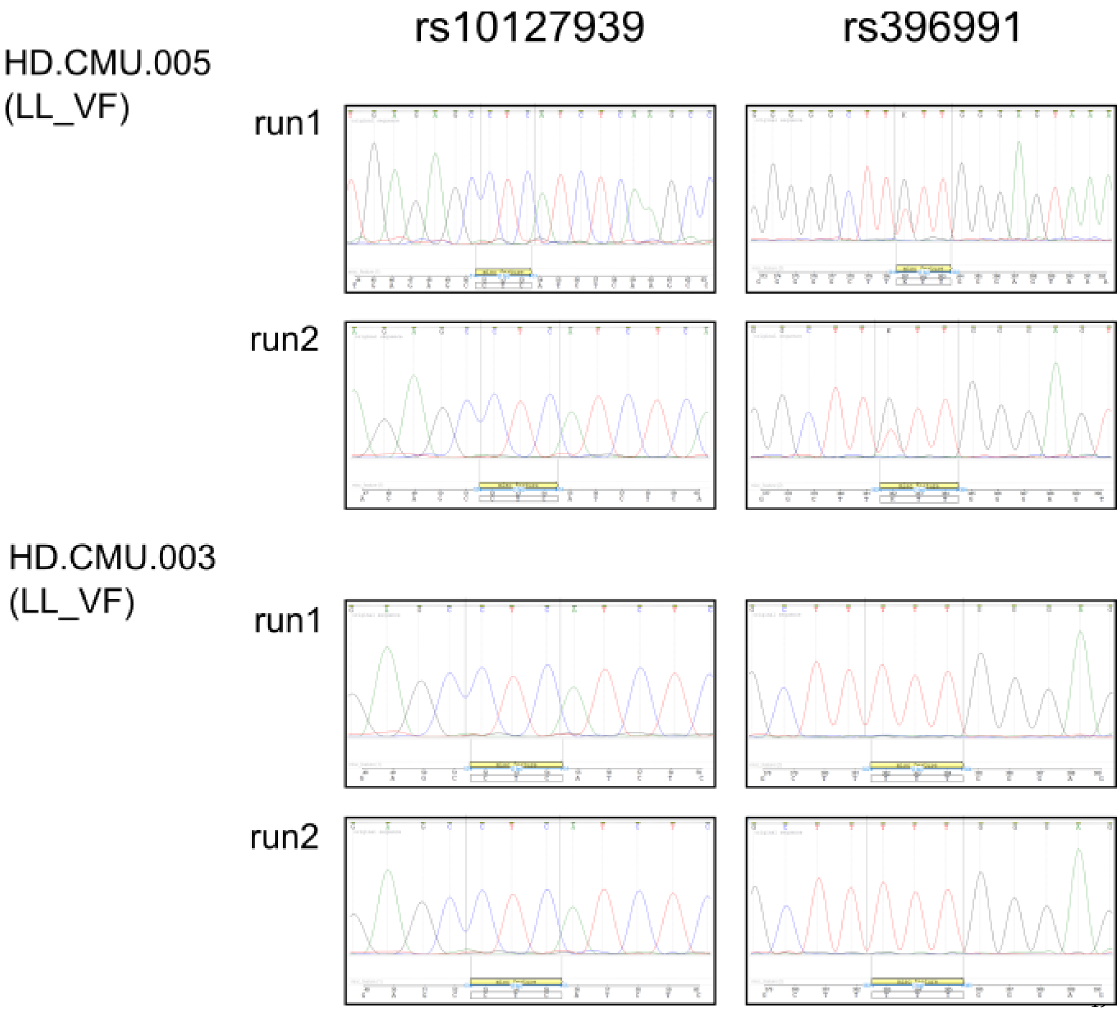

Figure S3. Sanger sequencing of the messenger RNA of *FCGR3A* coming from human NK cells.

Total RNA was extracted from freshly isolated NK cells purified from blood from two healthy donors, HD.CMU.003 and HD.CMU.005. Each RNA preparation was converted into cDNA and amplified by PCR on two separate occasions with primers region covering both SNPs, rs10127939 (L66H/R), left, and rs396991 (V176F), right. Amplicons were sent for Sanger sequencing using forward and reverse primers. Chromograms of each independent run are shown.

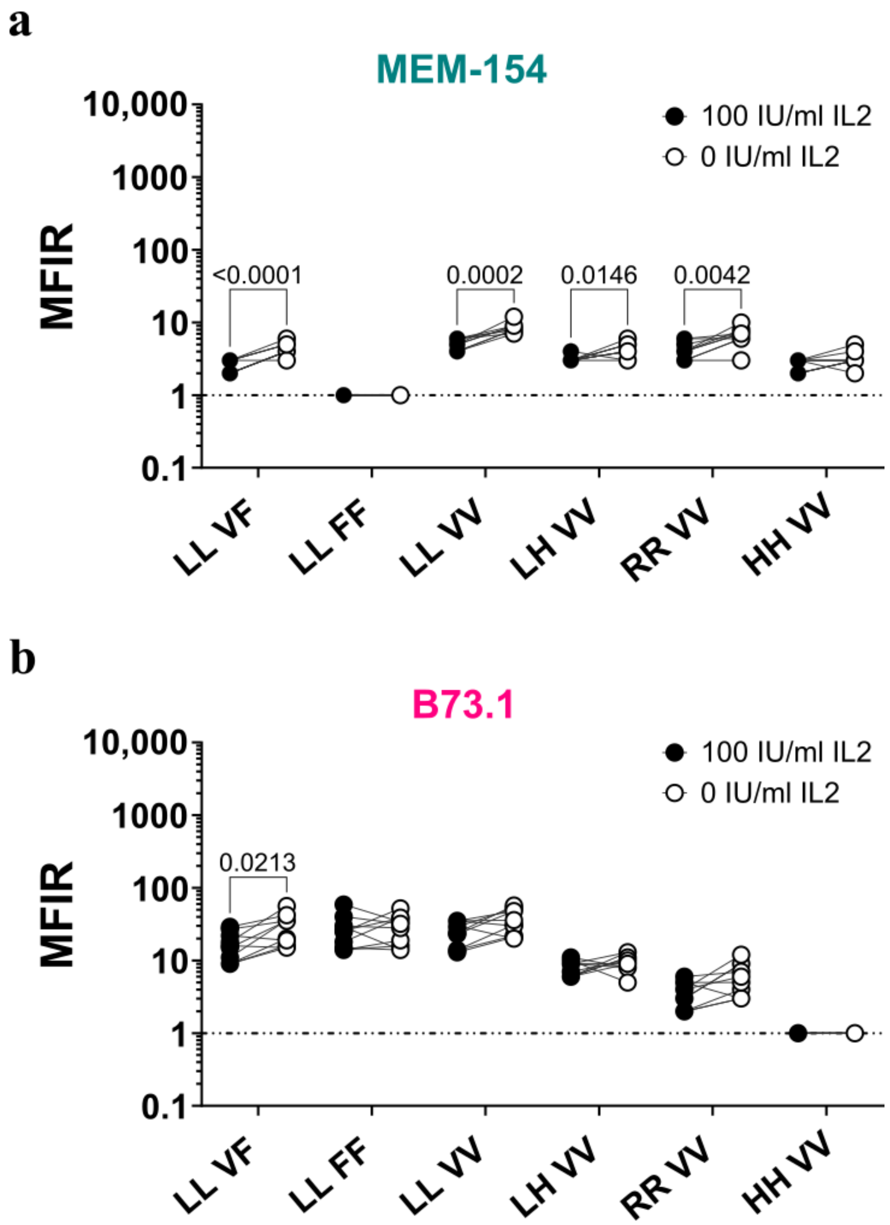

Figure S4. Clones B73.1 and MEM154 confirmed the findings of 3G8 for the increase of FcγRIIIa/CD16 due to the lack of interleukin-2 during overnight cultures**.**

Surface expression of FcγRIIIa/CD16 was analyzed by flow cytometry in NK-92 cell lines using flow cytometry. The cells were stained with anti-CD16 monoclonal antibodies, clones (**a**) MEM154 and (**b**) B73.1 after three days in culture with IL2 (black circles) or overnight IL-2-deprived (white circles). The plots show pooled data of the mean intensity ratio (MFIR) from six different NK-92 transfectants analyzed in 10 independent experiments, after three days in culture with 100IU/ml of IL2 or overnight IL2 deprivation. Comparisons were performed using ratio paired t-test. No values are shown if $P \geq 0.05$.

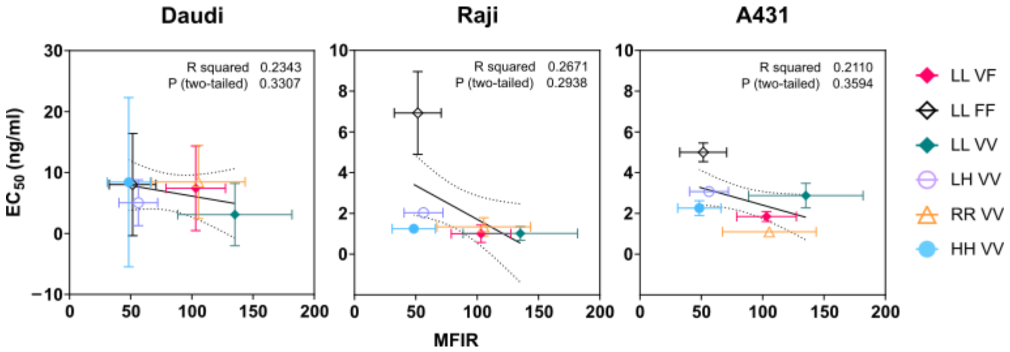

Figure S5. Correlation in NK-92 transfectants between the expression of FcγRIIIa/CD16 and antibody-dependent cell-mediated cytotoxicity (ADCC).

Flow cytometry was used to analyze the expression of FcγRIIIa/CD16 on various NK-92 cell lines. The clone 3G8 monoclonal antibody was used after the cell cultures were deprived overnight of interleukin 2. The level of expression is presented as the mean fluorescence intensity ratio (MFIR), which is calculated by dividing the mean fluorescence intensity of the sample by that of the matching isotype control. The ADCC evaluation of pooled data involved estimating the effective concentration required to achieve 50% cytotoxicity (EC_{50}) for six effector cell lines (NK-92 transfectants) against three different targets. The correlation plots display Pearson correlations expressed as R squared and fit goodness estimated by two-tailed *P*-value.

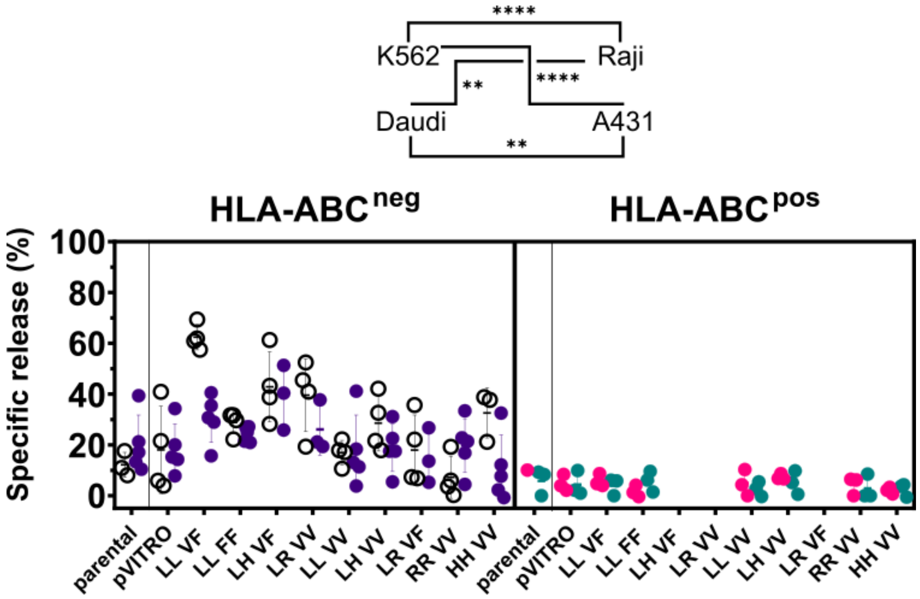

Figure S6. Comparison of direct cytotoxicity between target cells expressing or not HLA-ABC.

Non-radioactive cytotoxic assays were used to measure direct cytotoxicity specifically for K562 (open circle). For Daudi (puple), Raji (magenta), and A431 (green), values were extracted from the cytotoxicity controls in the ADCC assays. The target elimination is expressed as the mean ± SD of the percentage (%) of specific lysis in all cases. Overnight deprivation of IL-2 was necessary for all cell lines, including parental NK-92 cells and transfectants, before the assays. The effector-to-target (E:T) ratios were 5:1 for K562, Daudi, and Raji, and 10:1 for A431. The plot displays the direct cytotoxicity of targets that lack HLA-ABC on the left panel, including K562 (open circle) and Daudi (purple), and those that express it on the right panel, including Raji (pink) and A431 (green), for all effector cells analyzed. Each symbol represents an independent experiment. Comparisons between targets were performed using Dunnett's T3 multiple comparison test. Significance is indicated by **** for $P < 0.0001$; *** for $P < 0.001$; ** for $P < 0.01$; and * for $P < 0.05$.

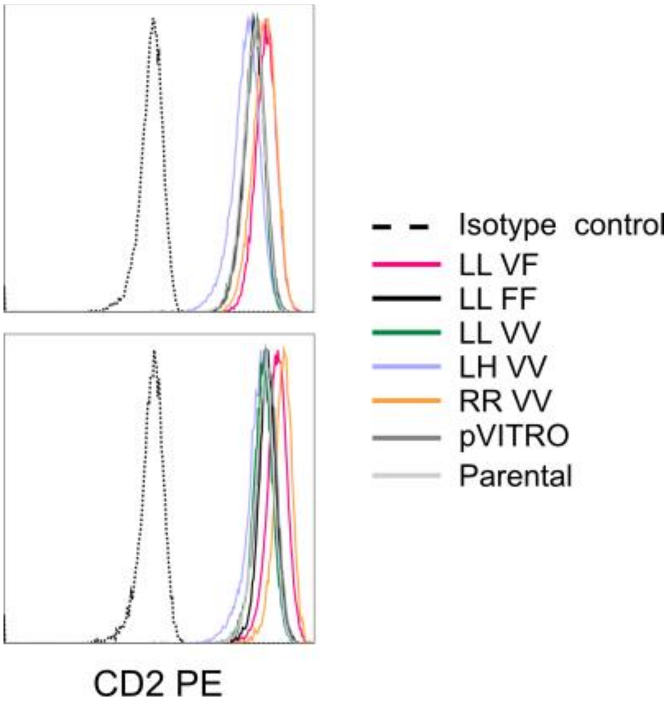

Figure S7. Expression of CD2 on NK-92 cell lines**.** Flow cytometry analysis for the expression of CD2 on NK-92 cell lines.

Cells were stained for 30min at 4 °C at previously titrated isotype control (dashed lines) and anti-human CD2 antibody conjugated to PE. Overlay histograms are shown for two independent determinations.
